# Chinese endemic medicinal plant *Bolbostemma paniculatum* (Maxim.) Franquet: A comprehensive review

**DOI:** 10.3389/fphar.2022.974054

**Published:** 2022-09-07

**Authors:** Yujiao Zhou, Junyu Liu, Jianqiong Zhang, Yi Xu, Wangni Li, Pang Gao, Yanghuan Xing, Lehong Huang, Xuhua Qin, Shenrui Jin

**Affiliations:** ^1^ College of Pharmacy, Chengdu University of Traditional Chinese Medicine, Chengdu, China; ^2^ School of Basic Medical Sciences, Chengdu University of Traditional Chinese Medicine, Chengdu, China; ^3^ Pediatric Department, Ya’an City Hospital of Traditional Chinese Medicine, Ya’an, Sichuan, China

**Keywords:** *Bolbostemma paniculatum* (Maxim.) Franquet, traditional application, botany, chemical components, pharmacological activities, quality control

## Abstract

*Bolbostemma paniculatum* (Maxim.) Franquet is a unique species in China with a long history of medicinal use, which has the effects of detoxifying, dissolving lumps and dispersing swellings. And it is commonly used to treat many diseases, such as carbuncle and sore, acute mastitis, mammary cancer, scrofula and subcutaneous nodule traditionally. Modern clinical studies have found that *B. paniculatum* and its compounds can be used for the treatment of a variety of cancers, mastitis, hyperplasia of mammary glands, chronic lymphadenitis, cervical lymph tuberculosis and surgical wart skin diseases, and the curative effect is positive. At present, a variety of Chinese patent medicines containing *B. paniculatum* have been exploited and marketed in China for the treatment of cancers, breast diseases and flat warts. This review article comprehensively discussed the traditional application, botany, chemical components, pharmacological activities, and quality control of *B. paniculatum*, put forward some noteworthy issues and suggestions in current studies, and briefly discussed the possible development potential of this plant as well as future research perspectives. 96 compounds have been isolated from *B. paniculatum*, including triterpenoids, sterols, alkaloids and other components, of which triterpenoid saponins are the main bioactive components. The crude extracts and monomer compounds of *B. paniculatum* have a wide range of pharmacological activities, such as anti-tumor, antiviral, anti-inflammatory, immunoregulatory, and so on. Moreover, its anti-tumor mechanism involves many aspects, including inhibiting cell proliferation, promoting cell apoptosis, blocking the cell cycle, interfering with cell invasion and metastasis, suppressing angiogenesis, and regulating autophagy. While there is a lack of systematic and in-depth research on its anti-tumor active components and mechanism of action at the moment; and a tight connection between the chemical composition and pharmacological activity of *B. paniculatum* has also not been established. Besides, a systematic quality determination standard for *B. paniculatum* should also be built, in order to carry out further research.

## 1 Introduction

*Bolbostemma paniculatum* (Maxim.) Franquet is an endemic species in China (Editorial Committee of flora of China, 1986), which belongs to the Bolbostemma genus of Cucurbitaceae family; and its traditional medicinal part is dry tuber (Wang et al., 2015). *B. Paniculatum* has a long history of medication in China, a large number of traditional applications have shown that it is able to eliminate the lumps in many parts of human body and can be used to treat many diseases, such as pyogenic infection and ulceration of skin, acute mastitis, mammary cancer, scrofula and subcutaneous nodule. Modern clinical studies have found that *B. paniculatum* and its compounds can be used for the treatment of a variety of cancers (Ling, 2015), mastitis, hyperplasia of mammary glands (Wang, 1995), chronic lymphadenitis, cervical lymph tuberculosis and surgical wart skin diseases (Xu et al., 1980); and the definite curative effect has been achieved.

So far, 96 compounds have been isolated from *B. paniculatum*, including triterpenoids, sterols, alkaloids and other components, of which triterpenoid saponins are the main bioactive components. The compounds isolated from *B. paniculatum* exhibit various pharmacological activities, such as anti-tumor, antiviral, anti-inflammatory, immunoregulatory, and so on.

*B. paniculatum* has received increasing attention because of its good medicinal values. After collecting and sorting out the research information of *B. paniculatum* from scientific journals and reports through library and electronic database (including PubMed, Science Direct, CNKI, Web of Science and Baidu Scholar), we comprehensively reviewed traditional application, the botany, chemical components, pharmacological activities, and quality control of *B. paniculatum*, in order to provide reference for further study.

## 2 Traditional applications

*B. paniculatum* known as “Tu Beimu” is a unique species in China with a long history of medicinal use. However, there were many kinds of drugs used in the name of “Beimu” in ancient China; and according to researches, at least 4 families, 6 genera, and more than 10 species of plants (Shang and Liu, 1995). There is a certain disorder of the basic source, while the common species are mainly three types: *Bolbostemma paniculatum* (Maxim.) Franquet, *Fritillaria cirrhosa* D.Don and *Fritillaria thunbergii* Miq. At present, it is believed that *B. paniculatum* is the earliest “Beimu” used in China from the pre-Qin Dynasty to the Qin and Han Dynasties (770 BC to 221 BC) (Qiu, 2016). Later, *Fritillaria thunbergii* Miq. known as “Zhe Beimu” appeared in the Wei and Jin Dynasties; from the Tang Dynasty to the late Ming Dynasty, *Fritillaria cirrhosa* D.Don known as “Chuan Beimu” and other varieties appeared. Until the Qing Dynasty, the three “Beimu” varieties were officially distinguished and used independently. In order to avoid disputes over the historical efficacy and evolution of *B. paniculatum*, we did not adopt the ancient documents before the Qing Dynasty in the data collection of this article.

The Qing Dynasty’s “Ben Cao Cong Xin” (1757) (Wu et al., 2013) and “Ben Cao Fen Jing” (1840) (Yao, 2015) recorded that *B. paniculatum* can treat “phlegm” and “toxin.” Traditional Chinese medicine holds that “phlegm” and “toxin” often interplay and form abnormal masses in the body, which related to various tumors, lymphadenopathy and other diseases in modern medicine (Lou et al., 2020; Xu et al., 2021). There are descriptions of the treatment of “Swelling-abscess of breast” (referring to acute suppurative mastitis) and “Ru-yan” (referring to mammary cancer (Liu L. L. et al., 2013) by *B. paniculatum* in the medical classics such as “De Pei Ben Cao” (1761) (Yan et al., 1994), “Ben Cao Gang Mu Shi Yi” (1765) (Zhao, 2017), “Xu Ming Yi Lei’an” (1770) (Wei and Huang, 1997), “Cao Cang Zhou Yi’an” (1924) (Cao and Liu, 2005). In “Wu Ju Tong Yi’an” (1798) and “Gu Jin Yi Che” (1908) (Huai, 1958), it was used to treat “Luo-li” (referring to multiple nodules or masses in the neck). Although people knew little about the mechanisms of *B. paniculatum* at that time, it was fully recognized that it has the effects of detoxifying, dissolving lumps and dispersing swellings, which was an effective drug to eliminate lumps in various parts of human body via a large number of clinical practices, see Table 1. Till modern times, traditional Chinese medical science has adopted *B. paniculatum* to treat multiple tumors, mastitis, hyperplasia of mammary glands, lymph node tuberculosis, lymphadenitis, skin warts disease (Fu et al., 1984) and so on, all of which have achieved good efficacy clinically. Nowadays, nine Chinese patent medicines containing *B. Paniculatum* have been developed and marketed in China. Among them, Tianfoshen Koufuye is an anti-tumor agent, which can be used for many malignant tumors, such as lung, esophageal, gastric, liver and breast cancer (Wang et al., 2018). Six Chinese patent medicines such as Rupishu Jiaonang (Zhang, 2001), Xiaoru Sanjie Jiaonang, Rupiqing Jiaonang, Rupiqing Pian (Ji, 2020), Ruzengning Jiaonang (Chinese Pharmacopoeia Commission, 2020), Rupishu Pian (Liu Y. et al., 2019) have been used to treat hyperplasia of mammary glands, mastitis, etc. Besides, Pifubing Xuedu Wan are used for nettle rash, eczema, acne, acne rosacea, ulcerative naevus, scabies, etc. (Xin et al., 2004). Tubeimu Zaogan Zhusheye can be injected intramuscularly or applied externally to treat flat warts (Shi, 2004).

**TABLE 1:** Traditional application of *Bolbostemma paniculatum* (Maxim.) Franquet.

Dynasty of ancient China	Classic medical books	Application	References
The Qing Dynasty (1757 AD)	Ben Cao Cong Xin	Treat phlegm and toxin in surgery	Wu et al. (2013)
The Qing Dynasty (1761 AD)	De Pei Ben Cao	Treat swelling-abscess of breast	Yan et al. (1994)
The Qing Dynasty (1765 AD)	Ben Cao Gang Mu Shi Yi	It can disperse carbuncle and toxin, and treat acute mastitis and mammary cancer	Zhao (2017)
The Qing Dynasty (1770 AD)	Xu Ming Yi Lei’an	Treat mammary cancer	Wei and Huang (1997)
The Qing Dynasty (1808 AD)	Gu Jin Yi Che	Eliminate scrofula	Huai (1958)
The Qing Dynasty (1840 AD)	Ben Cao Fen Jing	Treat phlegm and toxin in surgery	Wu (1809)
The Qing Dynasty (1924 AD)	Cao Cang Zhou Yi’an	Treat various carbuncle	Yao (2015)

## 3 Botany

**FIGURE 1:**
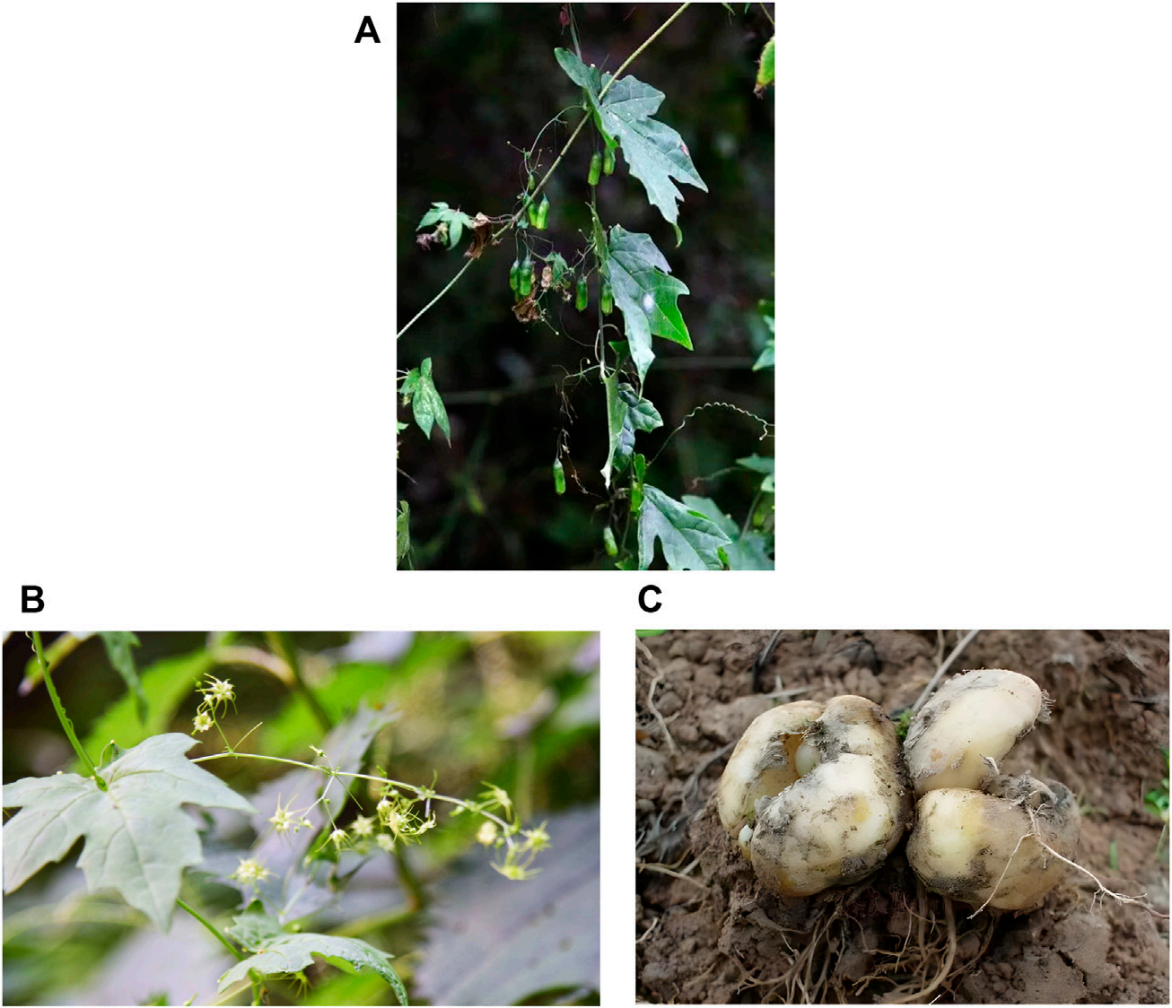
*Bolbostemma paniculatum* (Maxim.) Franquet: **(A)** Aboveground part, **(B)** flowers, and **(C)** stem tubers.

*B. paniculatum* is a perennial climbing herb belonging to the genus Bolbostemma of the Cucurbitaceae family. The tubers were hypertrophic, fleshy, and ivory; the stem is grassy, glabrous and climbing-like; its branch has prismatic grooves. The petiole is slender, about 1.5–3.5 cm long; the leaf blade is ovate-orbicular with 4–11 cm long and 3–10 cm wide, and is palmately 5-partite, there is 3–5 shallow lobes per lobe, the lateral lobes are ovate-oblong, acute, the central lobe is oblong-lanceolate, acuminate; there is a prominent gland at the tip of each basal lobe, and its leaves are glabrous or pilose only on veins. The cane vine is tendrils filiform, which grows momo or 2-fid. Flower are dioecious. The female and male inflorescences were evacuated panicles, with solitary flowers on it, and inflorescences are shaft filamentous, about 4–10 cm in length, the pedicel is slim, about 1.5–3.5 cm in length; the flowers are yellow-green, its calyx and corolla are similar, the segments are ovate-lanceolate and about 2.5 cm long, with filament tail at the top; there are five stamens which grow free; the filament apex is not enlarged with 0.3–0.5 mm long, the anthers is about 0.5 mm long, while the rostellum is on the back of anther and does not extend beyond the anthers. The ovary is ovoid with insignificant verrucous bulge, and it is 3-locular with two ovules in each locule, and there is three stylus which is 2-fid. The fruit is cylindric, about 1.5–3 cm in length and 1–1.2 cm in width, it cracks from top cap after ripening, the carpostegium is conic and has six seeds. Its seeds are ovate rhomboid and crineous, the surface has embossed bulge and irregular teeth on the edge, about 8–10 mm in length, 5 mm in width, 1.5 mm in depth, there is a long membranous wing at apex with 8–10 mm long. The flowering period is from June to August; the fruiting period is from August to September. The aboveground parts and tubers are shown in Figure 1. *B. paniculatum* is mainly produced in Hebei, Shandong, Henan, Shanxi, Shaanxi, Gansu, Eastern and southern Sichuan regions, and Northwest Hunan. It was born on the slope of the scrotum; and now has been widely cultivated (Editorial Committee of flora of China, 1986).

## 4 Chemical components

**FIGURE 2:**
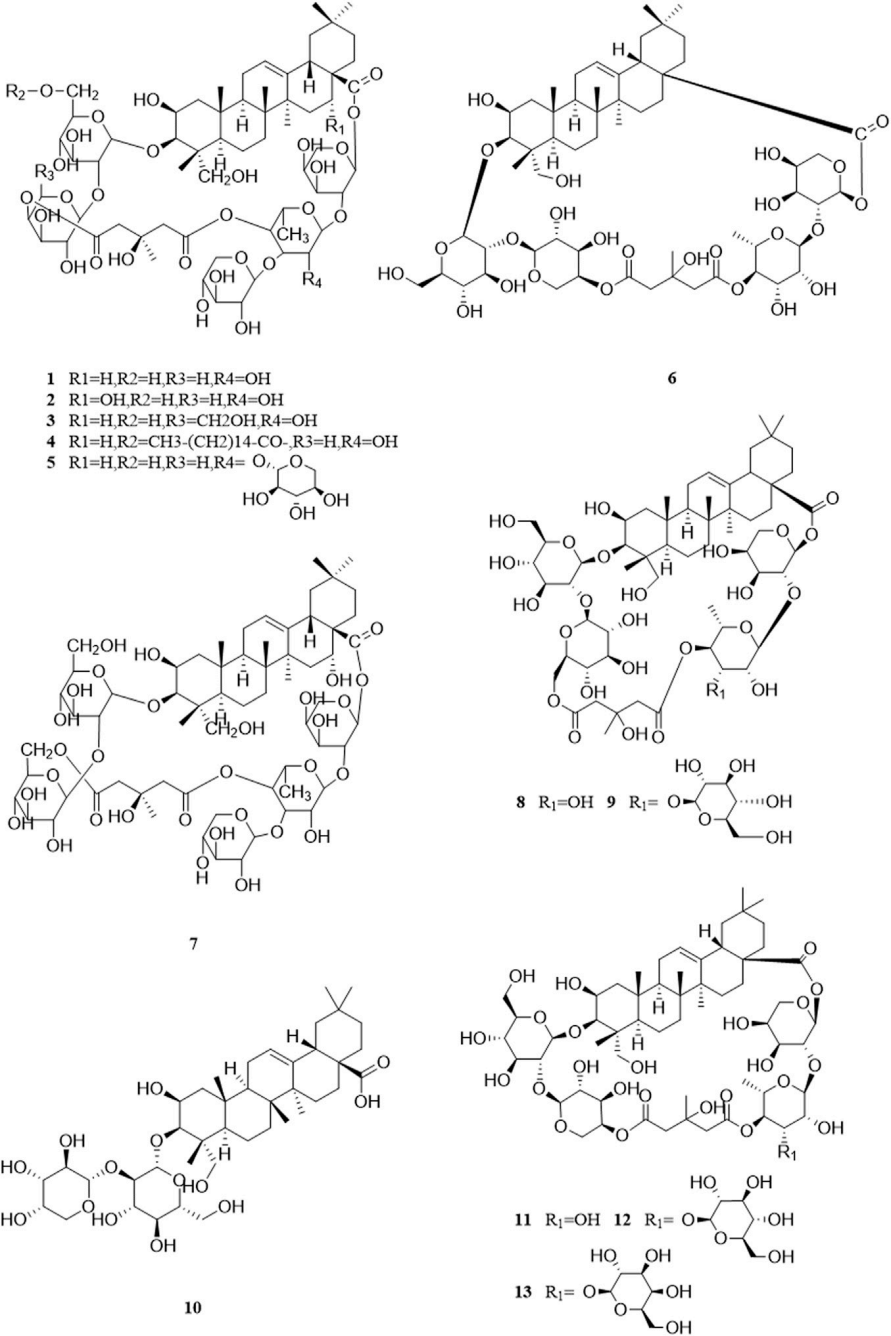
Structure of triterpenoids from *Bolbostemma paniculatum* (Maxim.) Franquet.

**FIGURE 3:**
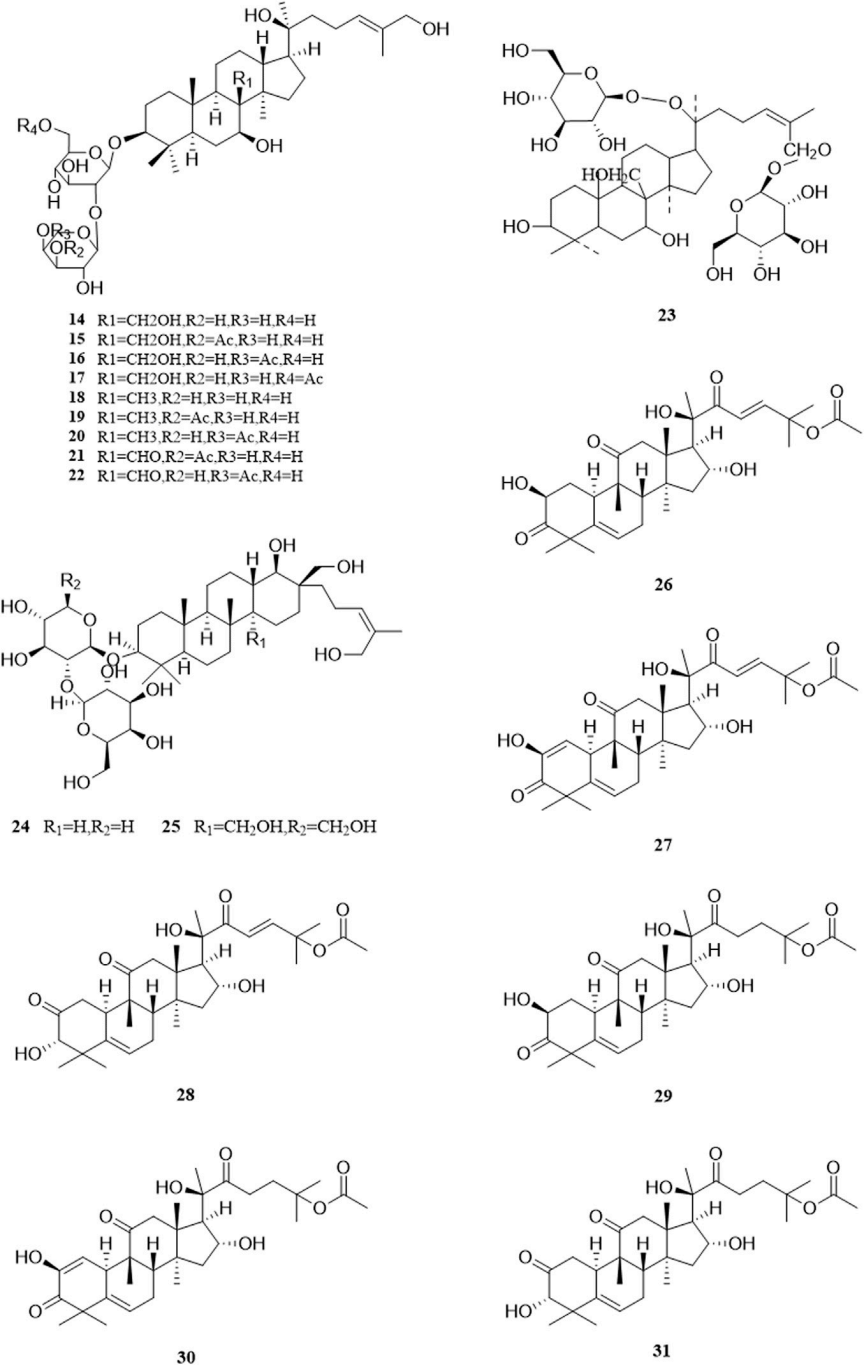
Structure of triterpenoids from *Bolbostemma paniculatum* (Maxim.) Franquet.

**FIGURE 4:**
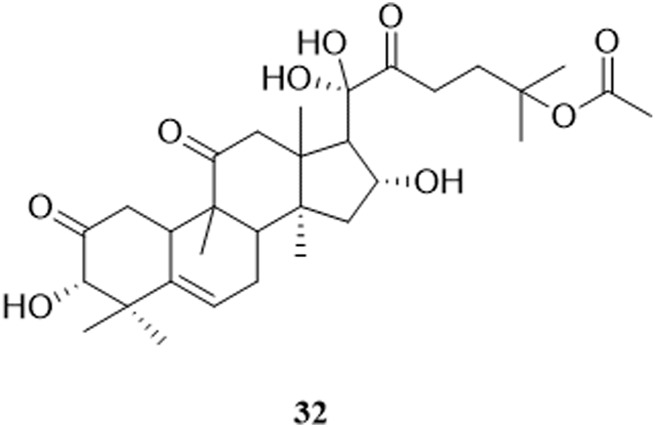
Structure of triterpenoids from *Bolbostemma paniculatum* (Maxim.) Franquet.

**FIGURE 5:**
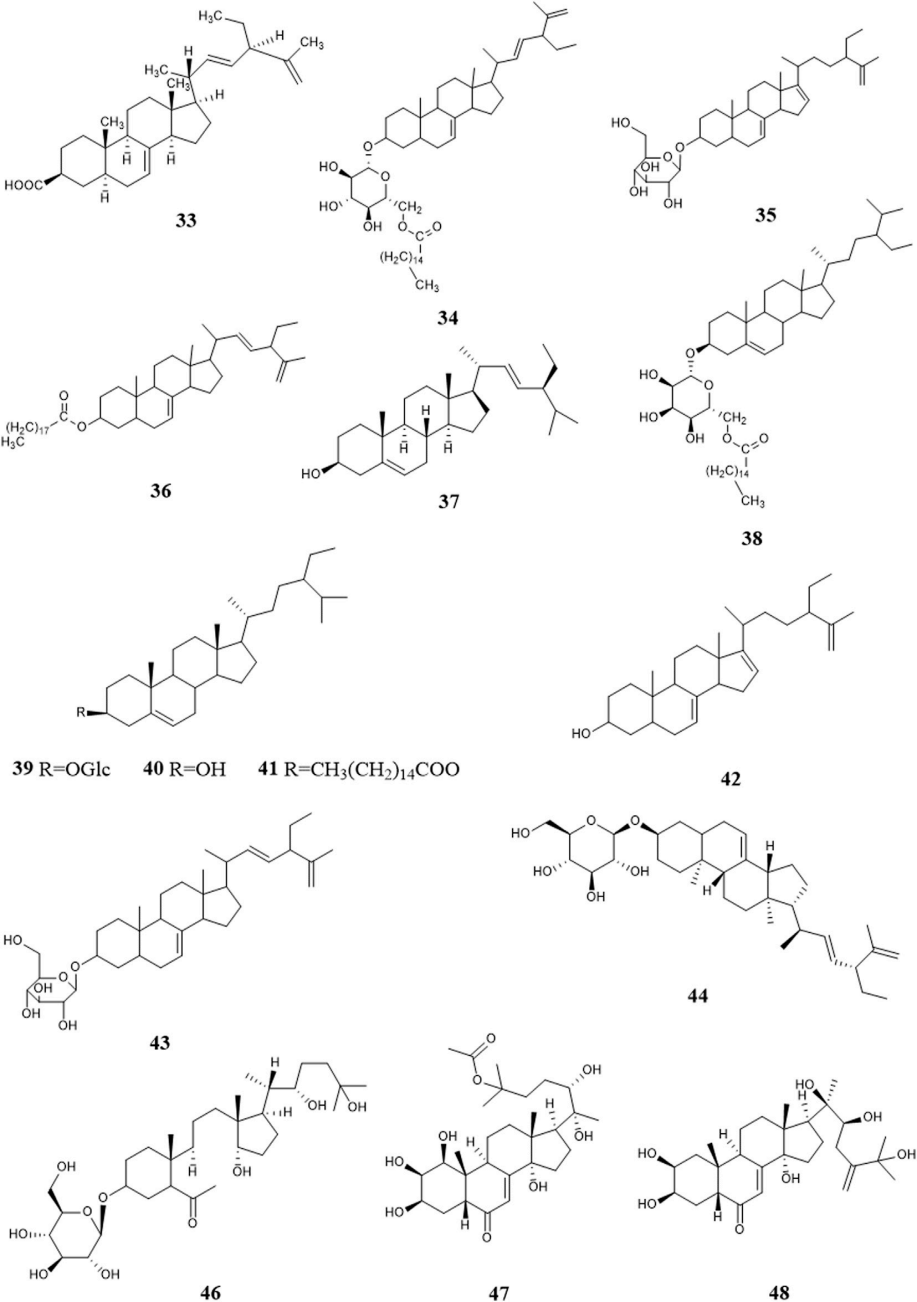
Structure of sterols from *Bolbostemma paniculatum* (Maxim.) Franquet.

**FIGURE 6:**
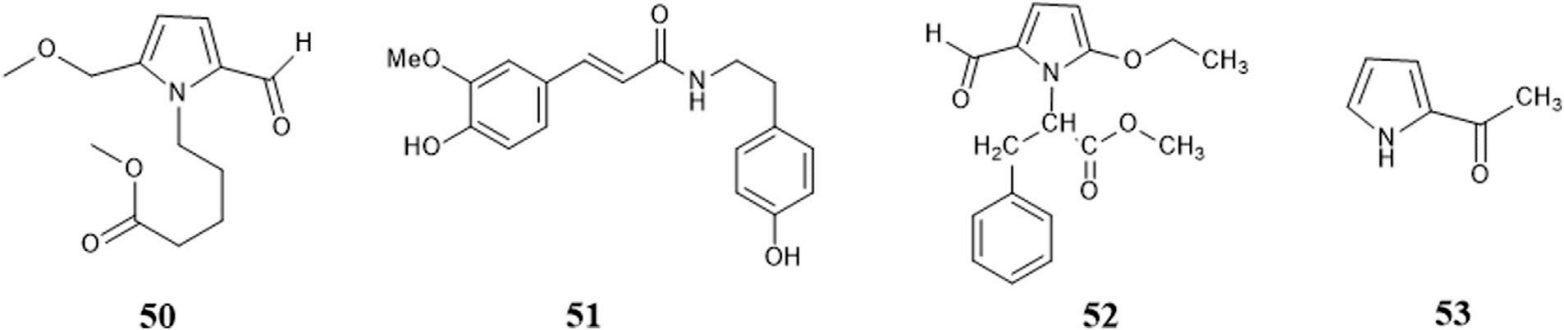
Structure of alkaloids from *Bolbostemma paniculatum* (Maxim.) Franquet.

**FIGURE 7:**
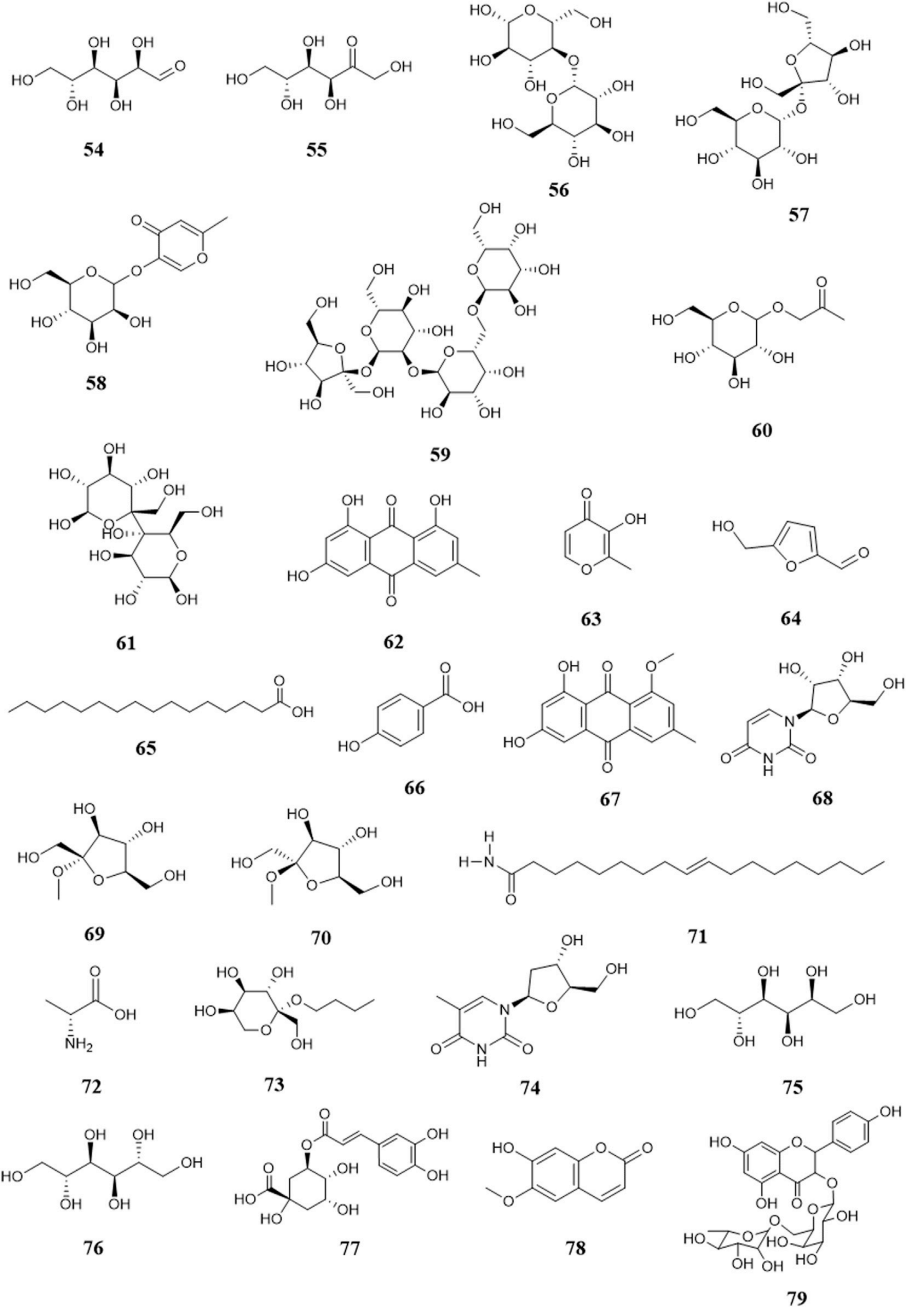
Structure of other compounds from *Bolbostemma paniculatum* (Maxim.) Franquet.

**FIGURE 8:**
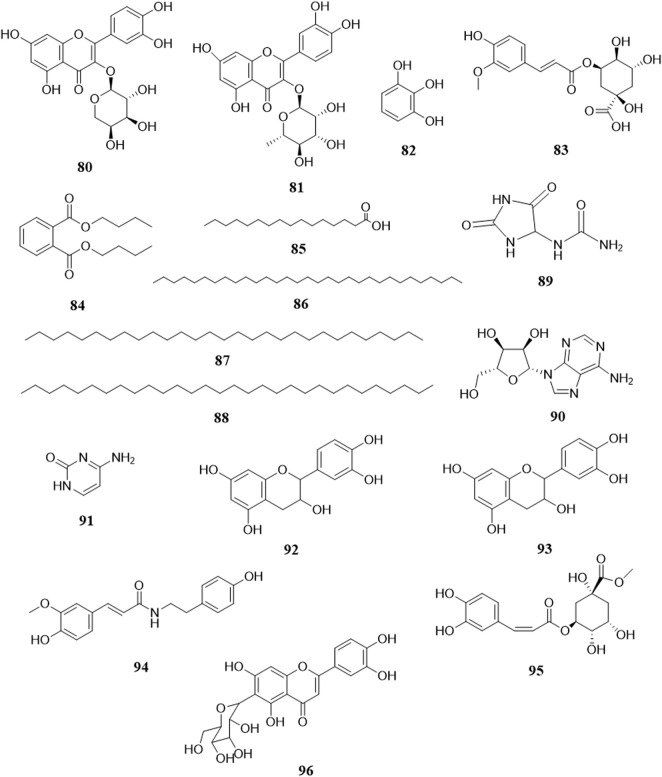
Structure of other compounds from *Bolbostemma paniculatum* (Maxim.) Franquet.

So far, 96 compounds have been isolated and identified from *B. paniculatum*, including triterpenoids (1–32), sterols (33–49), alkaloids (50–53) and other compounds (54–96). Among them, triterpenoids and their glycosides are the most important active components of *B. paniculatum*; the categories, names and corresponding structures of these compounds are summarized in Figures 2–8 and Supplementary Table S1.

### 4.1 Triterpenoids

Triterpenoids, which are a large group of constituents found in *B. paniculatum*, are one of the major active constituents. Tubeimoside I, which is the most studied compounds with high content in *B. paniculatum*, it has curative effects on glioblastoma, pancreatic cancer, cervical carcinoma and condyloma acuminatum, and is one of the anti-tumor and antiviral active ingredients (Li and Wang, 2006). Triterpenoid saponins from *B. paniculatum* include 13 pentacyclic triterpenoids (**1**–**13**), which are Tubeimoside I (**1**), Tubeimoside II (**2**), Tubeimoside V (**3**), 6′-*O*-palmitoyltubeimoside I (**4**), Dexylosyltubeimoside III (**5)**, Lobatoside C (**6**), Tubeimoside III (**7**), Actinostemmoside E (**8**), Actinostemmoside F (**9**), Actinostemmoside H (**10**), Cucurbitacin B (**11**), Cucurbitacin E (**12**), Isocucurbitacin B (**13**); and 19 tetracyclic triterpenoids (**14**–**32**), which are Tubeimoside IV (**14**), 7β,18,20,26-tetrahydroxy-(20*S*)-dammar-24E-en-3-*O*-α-L-(3-acetyl)arabinopyranosyl-(1→2)-β-D-glucopyranoside (**15**), 7β,18,20,26-tetrahydroxy-(20*S*)-dammar-24E-en-3-*O*-α-L-(4-acetyl)arabinopyranosyl-(1→2)-β-D-glucopyranoside (**16**), 7β,18,20,26-tetrahydroxy-(20*S*)-dammar-24E-en-3-*O*-α-L-arabinopyranosyl-(1→2)-β-D-(6-acetyl)-glucopyranoside (**17**), 7β,20,26-trihydroxy-(20*S*)-dammar-24E-en-3-*O*-α-L-arabinopyranosyl-(1→2)-β-D-glucopyranoside (**18**), 7β,20,26-trihydroxy-(20*S*)-dammar-24E-en-3-*O*-α-L-(3-acetyl)arabinopyranosyl-(1→2)-β-D-glucopyranoside (**19**), 7β,20,26-trihydroxy-(20*S*)-dammar-24E-en-3-*O*-α-L-(4-acetyl)arabinopyranosyl-(1→2)-β-D-glucopyranoside (**20**), 7β,20,26-trihydroxy-8-formyl-(20*S*)-dammar-24E-en-3-*O*-α-L-(3-acetyl)arabinopyranosyl-(1→2)-β-D-glucopyranoside (**21**), 7β,20,26-trihydroxy-8-formyl-(20*S*)-dammar-24E-en-3-*O*-α-L-(4-acetyl)arabinopyranosyl-(1→2)-β-D-glucopyranosid (**22**), 23,24-dihydrocucurbitacin E (**23**), 23,24-dihydrocucurbitacin B (**24**), 23,24-dihydroisocucurbitacin B (**25**), Isocucurbitacin D 25-*O*-acetate (**26**), 3-*O*-α-L-arabinopyranosyl-(1→2)-β-D-glucopyranosyl-bayogenin-28-*O*-β-D-xylopyranosyl-(1→ 3)-α-L-rhamnopyranosyl-(1→2)-α-L-arabinopyranosyl ester glycosides (**27**), Lobatoside A (**28**), Lobatoside B (**29**), Lobatoside E (**30**), Lobatoside D (**31**), Lobatoside F (**32**). It was found that the cytotoxic activity of the bulbs of *B. paniculatum* mainly came from cucurbitacine triterpenoid sapogenins (**12**, **13**, **23**, **25**) and the cyclic bisdesmosides (**1**, **3**, **5**, **6**); structurally, the macrocyclic structure of cyclic bisdesmosides plays an important role in their potential cytotoxicity and has certain anticancer potential (Tang et al., 2015). These compounds and their corresponding structures are shown in Figures 2–4 and Supplementary Table S1.

### 4.2 Sterols

Sterols are also one of the major active constituents of *B. paniculatum*. Liu et al. (2004b) isolated four sterols from the chloroform extract, which are Stigmasta-7,22,25-triene-3-ol (**33**), Stigmasta-7,22,25-triene-3-*O*-nonadecanoic acid ester (**34**), Stigmasta-7,22,25-triene-3-*O*-β-D-(6′-palmitoyl)glucopyranosid e (**35**), Stigmasta-7,22,25-triene-3-*O*-β-D-glucopyranoside (**36**). Zheng et al. (2005a) used solvent method and various chromatographic methods (silica gel column chromatography, dextran gel column chromatography, HPLC, etc.) to get Stigmasterol (**37**), Daucosterol palmitate (**38**), Daucosterol (**39**), β-sitosterol (**40**), β-sitosterol palmitate (**41**); and other studies isolated Stigmasta-7,16,25-triene-3-ol (**42**), Stigmasta-7,16,25-triene-3-*O*-β-D-glucopyranoside (**43**), (3β,22E)-stigmasta-7,22,25-trien-3-yl-β-D-glucopyranoside (**44**), Uzarigenin-3-β-sophoroside (**45**), Sileneoside H (**46**), Integristerone A-25-acetate (**47**), 24(28)-dehydromakisterone A (**48**), 3-oxo-androsta-1,4-dien-17a′-spiro-2′-3′-oxo-oxetane (**49**). Liu Y. G. et al. (2013) isolated and extracted the fat-soluble components of fresh *B. paniculatum*, which mainly contained sterols, and found that these components have cytotoxic effects on some cancer cell lines and have certain anti-tumor activity. These compounds and their corresponding structures are shown in Figure 5 and Supplementary Table S1.

### 4.3 Alkaloids

Zeng Y. et al. (2018) used UPLC/LTQ-Orbitrap MSn analysis and multivariate statistical analysis to quickly identify the medicinal material of *B. paniculatum*, and isolated three alkaloids: 4-(2-formyl-5-methoxymethylpyrrol-1-yl)butyric acid methyl ester (**50**), (E)-*N*-hydroxy phenyl ethyl-3-(4-hy-droxy-3-methoxy phenyl) acrylamide (**51**); in addition, Liu et al. (2003) isolated and structurally identified two new pyrrole alkaloids, which are 2-(2-formyl-5-methoxymethylpyrrol-1-yl)-3-phenylpropionic acid methyl ester (**52**), α-methyl pyrrole ketone (**53**). These compounds and their corresponding structures are shown in Figure 6 and Supplementary Table S1.

### 4.4 Others

There are few sugars in *B. paniculatum*, mainly maltose and sucrose. The tubers of *B. paniculatum* contain maltose, after drying, it contain sucrose; the petiole mainly contains reducing sugar, and the leaves mainly contain sucrose (Zhao et al., 2010); including Glucose (**54**), D-fructose (**55**), Maltose (**56**), Sucrose (**57**), Stachyose (**58**). Other compounds with important biological activities have also been found in different parts of *B. paniculatum*, such as glycosides: Isomaltol mannoside (**59**), α-hydroxyacetone glucoside (**60**), β-D-glucose 2→1 β-D-glucoside (**61**), Methyl α-D-fructofuranoside (**62**), Methyl β-D-fructofuranoside (**63**), n-Butyl-β-D-fructopyranoside (**64**); anthraquinones: Emodin (**65**), Emodinmonomethylether (**66**); flavonols: Quercitrin (**67**), 3-*O*-[β-D-pyranrham-nose-(1-6)-β-D-galactopyranose]-5,7,4′-trihydroxyl flavone (**68**), 6-C-glucose-5,7,3′,4′-hydroxy flavone (**69**), Quercetin-3-*O*-α-L-arabinopyranoside (**70**); aromatic compounds: Maltol (**71**), 4-Hydroxybenzoic acid (**72**), Dibutyl phthalate (**73**); aldehydes: 5-hydroxymethylfurfural (**74**); fatty acids: Palmitic acid (**75**), Hexadecanoic acid (**76**), 9-octadecenamide (**77**), D-alanine (**78**); nucleosides: Uridine (**79**), Thymidine (**80**), Adenosine (**81**), Cytosine (**82**); alcohols: D-Sorbitol (**83**), D-Mannitol (**84**); phenols: Chlorogenic acid (**85**), Pyrogallol (**86**), Catechin (**87**), Epicatechin (**88**); phenylpropionic acid compounds: Scopoletin (**89**), 5-*O*-feruloylquinic acid (**90**); alkanes: n-Hentriacontane (**91**), Nonacosane (**92**), Triacontane (**93**); heterocyclic compounds: Allantoin (**94**); amides: (*E*)-N-(4-hydroxyphenethyl)-3-(4-hydroxy-3-methoxyphenyl)-acrylamide (**95**) and lignin compounds: (*Z*)-3-*O*-caffeoyl-4-*O*-methylquinic acid methyl ester (**96**). These compounds and their corresponding structures are shown in Figures 7, 8 and Supplementary Table S1.

## 5 Pharmacological activities

**FIGURE 9:**
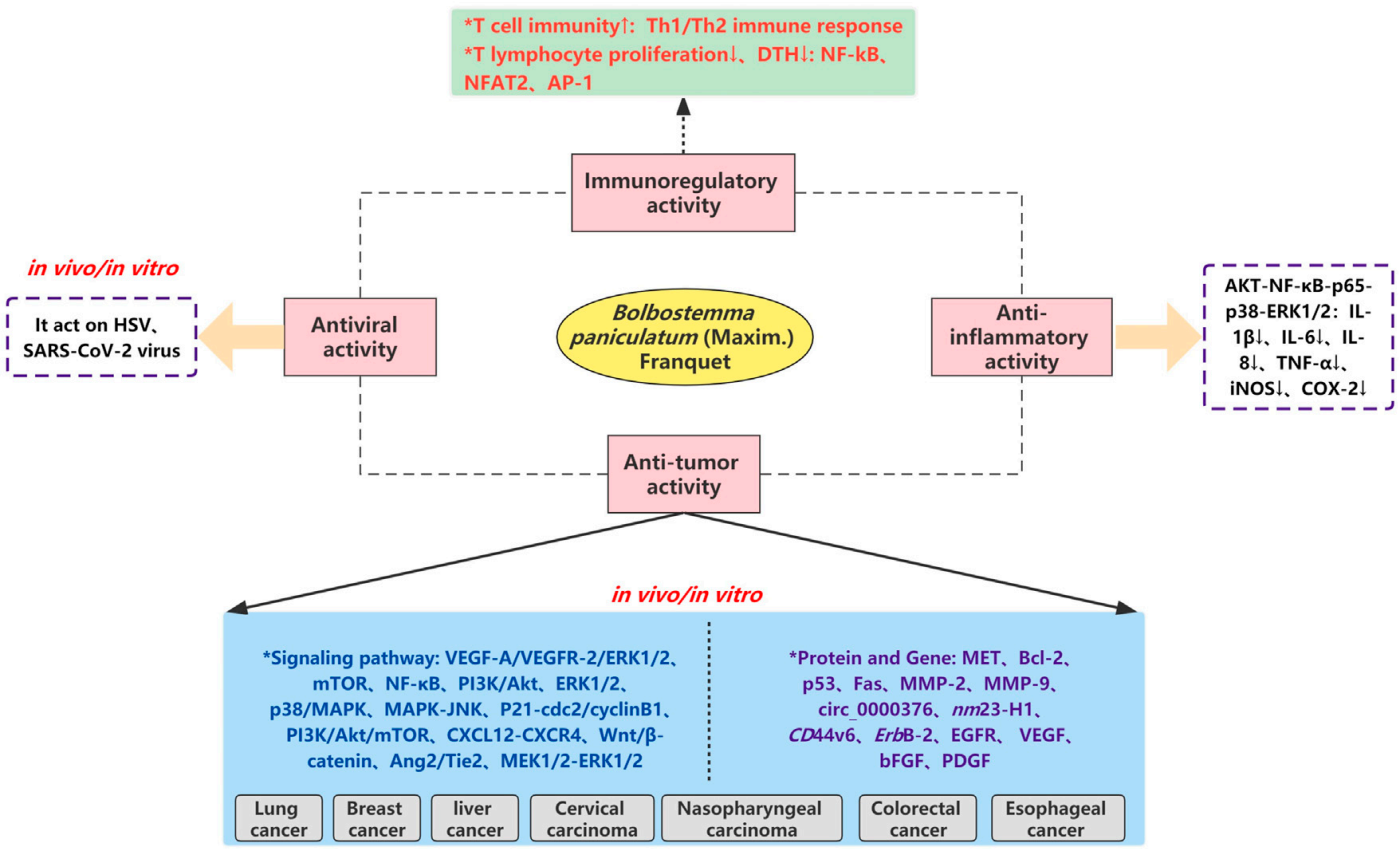
The molecular mechanism and pharmacological relationship network of *Bolbostemma paniculatum* (Maxim.) Franquet.

At present, modern pharmacological studies of *B. paniculatum* mainly focus on the anti-tumor activities of crude extracts and monomer compounds, and there are few relative studies on other pharmacological effects. In this part, we will comprehensively review the modern pharmacological effects of *B. paniculatum*, including anti-tumor, antiviral, anti-inflammatory, immuneoregulatory, etc.; detailed pharmacological activities and biological studies are shown in Figure 9 and Supplementary Table S2.

### 5.1 Anti-tumor activity

*B. paniculatum* can be used to treat various cancers in clinic. A large number of experimental studies have shown that the active compounds isolated from *B. paniculatum*, especially TBMS I, has anti-cancer effect on many kinds of cancer cell lines. TBMS I can exert its anti-tumor activity by inhibiting cell proliferation, directly exerting cytotoxic activity, inducing apoptosis, blocking the cell cycle, interfering with cell invasion and metastasis, repressing angiogenesis and regulating autophagy.

#### 5.1.1 Effect on proliferation

A prominent feature of tumorigenesis is abnormal cell proliferation. Several scholars have found that *B. paniculatum* exerts a certain inhibitory effect on the proliferation of prostate cancer PC3 cells (Wang et al., 2016), human liver cancer HepG2 cells (Wang et al., 2011), human ovarian cancer A2780/DDP cells (Liu et al., 2011), lung cancer NCI-H1299 cells, and glioblastoma cells. TBMS I can inhibit the proliferation of lung cancer NCI-H1299 cells by depressing the activity of the VEGF-A/VEGFR-2/ERK1/2 signaling pathway (Shi H. B. et al., 2018). In addition, TBMS I also increases the ubiquitination level of protooncogene (MET) to decrease the protein level of MET, thereby repressing the abnormal activation and amplification of MET, and ultimately inhibiting the proliferative activity of glioblastoma (Cao et al., 2019).

#### 5.1.2 Cytotoxic activity

A variety of chemical components of *B. paniculatum*, such as TBMS I, have direct cytotoxic activity, which can inhibit or kill cancer cells. Liu Y. G. et al. (2013) found that sterols were the main components of the fat-soluble extract of *B. paniculatum*; and *in vitro* experiment demonstrated that it have cytotoxic effects on four breast cancer cells (BT-549, MCF-7, MCF-7/ADR-RES, MDA-MB-231/ATCC). Tang et al. (2015) investigated the structure-activity relationship of triterpenoid saponins and their relationship with cytotoxicity in the bulbs of *B. paniculatum*, the results showed that TBMS I had moderate cytotoxicity against BCG-823, HeLa, HT-29, and MCF-7. Lin et al. (2016) found that the cytotoxic mechanism of TBMS I on NCI-H460 cells involved nucleolar stress-induced p53/murine double minute clone 2 (MDM2), mTOR, and NF-κB signaling pathways. Xu et al. (2009) discovered that the cytotoxic effect of TBMS I on HeLa cells was caused by mitochondrial dysfunction and the ER stress cell death pathway; through further study, they noticed that TBMS I-induced cytotoxicity involves multiple aspects, including molecular changes in ROS production, Ca^2+^ regulation, and G2/M cell cycle regulatory proteins (Xu et al., 2011).

#### 5.1.3 Effects on apoptosis and autophagy

Apoptosis is a special type of cell death, and tumorigenesis is often associated with the inhibition of apoptosis. Guo et al. (2006) found that embolization of Tubeimosides microcapsules can cause ischemia and hypoxia in tumor tissue of liver cancer, and result in necrosis and apoptosis; medicated serum of *B. paniculatum* decoction can induce apoptosis of human lung cancer A549 and H1299 cells (Mi et al., 2021). Chao et al. (2019) discovered that peiminine isolated from *B. paniculatum* can induce apoptosis in human hepatocellular carcinoma HepG2 cells through both extrinsic and intrinsic apoptotic pathways. Modern pharmacological studies have demonstrated that TBMS I shown a powerful ability in inducing apoptosis; and it can induce apoptosis of A549, PC9, SKOV-3, CNE-2Z, BGC823, DU145, EC109, HL-60, SW480, U251, choriocarcinoma cells, etc. According to the studies of several scholars on the apoptotic mechanism of TBMS I, they found that TBMS I-induced apoptosis in tumor cells is a complex event which is polygenic and multichannel. TBMS I can regulate apoptosis of ovarian cancer SKOV-3 cells (Chen et al., 2012), squamous esophageal carcinoma EC109 cells (Xu et al., 2013), and human prostate cancer DU145 cells (Yang et al., 2016) via two important pathways: endoplasmic reticulum stress and mitochondrial dysfunction. One of the major events in apoptosis involving mitochondrial dysfunction is the alteration of apoptotic protein expression; TBMS I promotes apoptosis by regulating the expression of multiple apoptotic proteins, such as Bcl-2 family, p53 protein, Fas protein, etc. It has been found that the mechanism of TBMS I-induced apoptosis of various cells is mainly to downregulate the expression of Bcl-2 and upregulate the expression of Bax, including choriocarcinoma cells (Huang et al., 2011), ovarian cancer SKOV-3 cells (Chen et al., 2012), human nasopharyngeal carcinoma CNE-2Z cells (Weng et al., 2003), lung cancer A549 cells (Zhang X. H. et al., 2011; Hao et al., 2015), gastric cancer BGC823 cells (Zhang et al., 2013), human anterior adenocarcinoma DU145 cells (Yang et al., 2016), human glioma U251 cells (Jia et al., 2015; Cao et al., 2020), etc. The study of Yu et al. (2006) discovered that TBMS I could also downregulate the expression of p53 and upregulate the expression of Fas to induce apoptosis of human rectal cancer SW480 cells. Furthermore, scholars also found that apoptosis refers to a variety of signal pathways, including PI3K/Akt, ERK1/2, p38/MAPK, MAPK-JNK, P21-cdc2/cyclinB1 signal pathways, etc.

As the basic physiological process of cells, the functional status of autophagy directly affects tumorigenesis and its treatment. TBMS I can induce the autophagy of breast cancer MDA-MB-231 cells, and its effect is achieved by regulating the activity of PI3k-Akt-mTOR signaling pathway (Liu F. F. et al., 2019). Similar studies have shown that TBMS I can also activate AMPK signaling pathway to induce autophagy in human hepatoma HepG2 cells (Ruan et al., 2020). On the one hand, it can play its role via AMPK signal pathway; on the other hand, it can also restrain lysosomal cathepsin to block autophagy flux, and act on colorectal cancer CRC cells (Yan et al., 2019), cervical cancer cells (Feng et al., 2018), and lung cancer cells (Wang et al., 2020) according to these two ways. Besides, Du et al. (2020) found that TBMS I could trigger the interruption of partial autophagy flux and cytoprotective autophagy in melanoma A375 cells, which was realized by over-activating MEK1/2-ERK1/2 cascade.

#### 5.1.4 Effect on cell cycle

Han et al. (2009) confirmed that the *B. paniculatum* preparation can block the transformation from G_1_ phase cells to S phase cells, which led to a relative increase of cells in G2/M phase; thus the growth of human tongue cancer Tca8113 cells was inhibited. Hu Z. et al. (2003) found that TBMS I blocks the cell cycle of human myeloid leukemia HL-60 cells by reducing the expression level of cyclin B1 related to the cell cycle. Besides, TBMS I can arrest cervical cancer HeLa cells in the G_2_/M phase, resulting in a significant inhibitory effect on its growth (Zhang et al., 2010). In the process of inhibiting the growth of human hepatoma HepG2 cells by TBMS II, the cells number in the G2/M phase increased; whereas the cells number in the G_0_/G_1_ phase decreased significantly (Chao et al., 2012).

#### 5.1.5 Intervention in invasion and metastasis

Invasion and metastasis of tumor cells is one of the significant biological characteristics of malignant tumors, which is the main cause of death for clinical cancer patients. The study of Bao et al. (2017) discovered that the dichloromethane extract of fresh *B. paniculatum* can inhibit the tumor growth of human triple negative breast cancer MDA-MB-231-GFP nude mice and the occurrence of lung metastasis; and the corresponding *in vitro* test results also confirmed its anti-metastasis effect (An et al., 2013). Tubeimosides inhibit the activity of PI3K/Akt/mTOR signaling pathway, thus inhibiting the migration ability of breast cancer MDA-MB-231 cells (Dou et al., 2019). TBMS I can also inhibit the invasion and metastasis of breast cancer MDA-MB-231 cells by downregulating the expression of CXCR4 or interrupting CXCL12-CXCR4 cell signal pathway (Peng et al., 2016). Similar studies have mentioned that TBMS I can notably inhibit the metastasis of OSCC cells (Wu et al., 2018). Related pharmacological studies have shown that TBMS I inhibits the invasion and metastasis of tumor cells by different signal pathways. First of all, it can inhibit Wnt/β-catenin signaling pathway to prevent invasion of human colorectal cancer SW480 and HCT-8 cells (Bian et al., 2015); secondly, it can also suppress the migration and invasion of non-small cell lung cancer NCI-H1299 cells by inactivating VEGF-A/VEGFR-2/ERK1/2 signaling pathway (Shi H. et al., 2018). In addition, there is a research found that TBMS I can exert certain inhibitory effects on the adhesion, invasion and migration abilities of human highly metastatic giant cell lung carcinoma PGCL3 cells (Yu et al., 2008a) and human hepatoma HepG2 cells (Zhong et al., 2016) by inhibiting the secretion and activity of MMP-2 and MMP-9. TBMS I represses the migration and invasion of cutaneous squamous cell carcinoma CSCC cells, the mechanism may be that it can downgrades circ_0000376, thereby promoting the expression of miR-203 (Wang et al., 2021). Through further study, it was found that TBMS I significantly inhibited the metastasis of B16 melanoma and Lewis lung cancer in mice; this effect was related to upregulating the expression of metastasis suppressor genes (*nm*23-*H*1) and downregulating the expression of metastasis promoting genes (*CD*44*v*6, *ErbB*-2) (Wang et al., 2006). Moreover, TBMS II can reduce the redox related oxidative stress, inhibit the activation of epidermal growth factor receptor (EGFR) and inhibit TGF-β1-induced multiple transfer steps, thereby inhibiting the adhesion, migration of human retinoblastoma cells and invasion (Chen et al., 2021).

#### 5.1.6 Antiangiogenesis

The growth, invasion and metastasis of solid tumors all depend on the formation of neovascularization; and TBMS I can curb tumor angiogenesis. Hu D. H. et al. (2003) preliminarily used angiogenesis model of chicken embryo chorioallantoic membrane to detect the effect of TBMS I on angiogenesis, and confirmed that it has a strong inhibitory effect on angiogenesis, and the dose-effect relationship is very obvious. The study of Yu et al. (2008b) shows that its antiangiogenesis mechanism is related to inducing apoptosis and inhibiting motility of vascular endothelial cells, and downregulating the expression of VEGF, bFGF, and PDGF. *In vitro* and vivo studies of Gu et al. (2016), TBMS I depresses angiogenesis based on its stimulating effect on the degradation of proteasomal VEGFR2 and Tie2, and downregulates the AKT/mTOR signaling pathway. Through further study, they found that the inhibitory mechanism of TBMS I against non-small cell lung cancer NCI-H4660 tumor vessel may be that it can significantly restrain the activation of VEGF/VEGFR2 and Ang2/Tie signal transduction pathways (Li T. Y. et al., 2016). Based on the inhibiting effect of TBMS I in HUVECs, Liu et al. (2020) speculated that the antiangiogenic effect may be related to the Piezo1 channel.

### 5.2 Antiviral activity

*In vitro* and *in vivo* tests showed that Tubeimosides had certain inhibitory effects on HSV1 virus and HBV virus (Zhou and Wu, 2005; Zhou et al., 2007; Zhang Y. et al., 2011). Wang et al. (2019) found that TBMS I can simultaneously inhibit the proliferation and genome replication of HSV1 and HSV2 viruses. In recent years, Ju et al. (2021) used high-throughput screening to identify that TBMS I showed obvious antiviral activity against SARS-CoV-2 infection *in vitro*.

### 5.3 Anti-inflammatory activity

*In vivo*, Yu et al. (2016) found that TBMS I can markedly inhibit PPDA-induced dermatitis in guinea pigs; and the study of Ma et al. (1991) shows that it could also significantly restrain TPA and AA-induced ear swelling in mice. In addition, TBMS I has a strikingly anti-inflammatory effect on rheumatoid arthritis (RA); Bao et al. (2018) and Liu et al. (2018) discovered it represses the production of pro-inflammatory cytokines as well as downregulate the expression of MMP-9. Zhang et al. (2018)’s research shows that TBMS I ameliorates PM_2.5_-induced lung injury in mice; and the mechanism may be that it reduce the level of inflammatory cytokines, such as IL-1β, IL-6, IL-8, TNF-α, so as to improve PM_2.5_-induced lung injury by inhibiting inflammation. Wu et al. (2013) utilize LPS-induced mice model of lung injury and LPS-stimulated RAW264.7 cells, and they found it remarkably inhibit the generation of pro-inflammatory cytokine (TNF-α, IL-6 and IL-1β) both *in vivo* and vitro. To some extent, TBMS I also has inhibitory effect on BV-2-mediated neuroinflammation; in tests on LPS-induced PD rat model LPS-exposed BV-2 cells, researchers confirmed that it could inhibit the activation of BV-2 and the reduction of dopaminergic neurons (He et al., 2018) *in vivo*; moreover, it probably works via inhibiting phosphorylation of AKT, NF-κB p65, p38 and ERK1/2, and then reduces the expression of pro-inflammatory mediators (iNOS, COX-2, TNF-α, IL-1β and IL-6).

Sepsis is a systemic inflammatory response syndrome caused by the invasion of pathogenic microorganisms such as bacteria into the body. Luo et al. (2020) found that the protective effect of TBMS I on sepsis mice was achieved by repressing the TLR4-MyD88-NF-κB-iNOS pathway; while similar pharmacological studies have shown that it may alleviate the pathological factors of sepsis-induced cardiac dysfunction and endothelial dysfunction (such as inflammation, oxidative stress, and apoptosis, etc.), which was raised by SIRT3 (Cheng et al., 2021; Yang et al., 2021).

### 5.4 Immunoregulatory activity

*B. paniculatum* is a promising immunoregulator. TBMS I can notably enhance antigen-specific humoral and cellular immunity responses, and lead Th1/Th2 immune responses in mice (Han, 2019). Liu et al. (2021)’s experiment demonstrated that TBMS I can also realize its anti-tumor effect by improving anti-tumor T cell immunity. *B. paniculatum* has inhibiting effect both on humoral and cellular immunity (Huang et al., 1992); the further test indicated that it markedly inhibit T lymphocyte proliferation *in vitro* and T cell-mediated delayed type hypersensitivity (DTH) *in vivo*, and the mechanism may be concerned in depressing the activation of NF-kB, NFAT2 and AP-1 signal transduction pathways (Huang et al., 1992).

### 5.5 Others

TBMS I has potential development value in the treatment of osteoporosis; it can inhibit NF-κB signaling pathway to reduce the production of osteoclasts, so as to protect against bone damaging diseases such as osteoporosis caused by type 2 diabetes (Yang et al., 2019; Yang et al., 2020). In addition, the extract of *B. paniculatum* also has certain antibacterial effect, researches confirmed that the 75% ethanol extract of *B. paniculatum* has obvious inhibitory action on Salmonellae, *S. agalactiae* and *Staphylococcus aureus*; while this action becomes inapparent on *Bacillus coli* (Chen et al., 2009). It has been reported that *B. paniculatum* possesses definite spermicidal efficacy, which affects the biomembrane system of sperm and damages the plasma membrane, acrosome and mitochondria of sperm; furthermore, its spermicidal active constituent are saponins. Lv et al. (2021) found that TBMS I has certain antioxidant activity and protects myocardial ischemia-reperfusion injury *via* SIRT3-dependent oxidative stress and apoptosis regulation.

## 6 Quality control

The current edition of Pharmacopoeia of the People’s Republic of China (Chinese Pharmacopoeia Commission, 2020) stipulates that the index component of *B. paniculatum* is TBMS I (C_63_H_98_O_29_); its content is determined by high-performance liquid chromatography (General rule 0512), and the content shall not be less than 1.0%; this product is calculated as dry product. Moreover, the identification, inspection and determination of *B. paniculatum* are also documented; it is required to use ethanol as the solvent and hot-dipped coating which belongs to determination of alcohol soluble extract method (General rule 2201), and shall not be less than 17.0%.

Traditional Chinese medicine (TCM) has the characteristics of multi-component and multi-target, so it is difficult to comprehensively judge the quality of *B. paniculatum* with only one component as the standard. With the continuous improvement of modern isolation and identification methods, several investigators have used a variety of methods to evaluate the quality of *B. paniculatum*; among them, HPLC is one of the most commonly used analytical methods, and it is also the main method for the analysis of compounds in *B. paniculatum*. In Wang et al. (2015)’s research, he used TBMS I and Tubeimosides as detection indicators, and adopt orthogonal test to evaluate and prioritize extracting craft. Wei et al. (2000) measured the content of TBMS I by HPLC, extracted it with water, and determined the content of *B. paniculatum* from different sources. Except the method for merely determining the content of TBMS I, there is another way to simultaneously measure the content of TBMS I, TBMS II, TBMS III for the first time (Huang et al., 2016). However, in order to describe and evaluate the quality of *B. paniculatum* as a whole, it is necessary to establish its fingerprints; Li et al. (2007) established its fingerprint via HPLC method, and they found that there are 18 common fingerprint peaks from 15 places of origin. Through further study, investigators built the characteristic maps of saponins in *B. paniculatum* from different habitats, and they discovered that the main components affecting the quality of the medicinal materials were TBMS I, TBMS II, TBMS III; at the same time, similarity evaluation, principal component analysis and cluster analysis are also applied, which can be used for the quality evaluation of *B. paniculatum* more comprehensively, accurately and effectively (Zeng Y. L. et al., 2018). Huang et al. (2020) utilize another method to determine its content, QAMS, and established the UPLC fingerprint of *B. paniculatum*; the results showed that there were four common peaks in the fingerprint of 19 batches of medicinal materials. In addition, Cui et al. (2008) set up a method to measure TBMS I in rat serum; because the amount of TBMS I in rat serum is very low, so the experiment took a solid-phase extraction method to process serum samples, which can rapidly, easily, and accurately determine the content of its preparation and serum. Other researchers used thin-layer chromatography (Wang et al., 1993; Ren and Zhao, 2005) and ultraviolet spectrophotometry (Ma and Yang, 2002) to evaluate the quality of *B. paniculatum*, but the accuracy was not as good as the above methods.

To sum up, the quality control of *B. paniculatum* has not been comprehensively and systematically studied. Therefore, it is necessary to further explore and establish a more objective and comprehensive quality standard, so as to achieve reliable, reasonable and systematic quality evaluation and quality control for *B. paniculatum*.

## 7 Discussion and prospect

*B. paniculatum* is a unique species in China with a long history of medicinal use; traditional Chinese medicine science believes that it has the effects of detoxifying, dissolving lumps and dispersing swellings, and can eliminate the lumps in many parts of human body. At present, doctors of TCM use it to treat many diseases such as various cancers, mastitis, breast hyperplasia, chronic lymphadenitis, cervical lymph node tuberculosis and surgical warts skin diseases, and achieve certain curative effects. Over the past 20 years, scholars have done a lot of researches on the chemical components and pharmacological effects of *B. paniculatum*, and have also obtained some research results; while there is still a long way to reveal its clinical efficacy.

After teasing out the traditional applications, botany, chemical composition, pharmacological activities, quality control, we propose the following considerations and suggestions:

Firstly, it is known that *B. paniculatum* contains many components such as saponins, sterols, alkaloids, etc.; but the researches on its active components mostly concentrated in the saponins (especially TBMS I), and the activity research of other components have not been involved. Meanwhile, its pharmacological studies are mostly limited to the study of anti-tumor activity; and its anti-tumor effects are mostly conducted *in vitro* model, but few *in vivo* experiments. Studies on its material basis of anti-tumor effects are not enough, especially the adequate correspondence between components and anti-tumor effect has not been established.

Secondly, some traditional applications of *B. paniculatum*, such as treating various wart diseases, have not been thoroughly studied in modern times. And its antiviral activity also has great research value, such as the inhibition of SARS-CoV-2 virus.

Thirdly, TBMS I can play an immunosuppressive role at a small dose, which is also an aspect worthy of attention.

Fourthly, the acute toxicity and long-term toxicity of *B. paniculatum* and its active components are rarely reported, so it is necessary to promote research in this area to establish its safety.

Fifthly, the 2020 edition of Chinese Pharmacopoeia only selects TBMS I as the quality control index, which is difficult to comprehensively evaluate its quality; hence, its systematic quality control methods need to be further explored.

In conclusion, *B. paniculatum* has a long history of application, which has exact efficacy and promising development prospect, so it does deserve in-depth studies from modern investigators. We hope that this paper can provide a valuable reference for those who are interested in the study of *B. paniculatum*.
