# Supplementary material for: Chinese endemic medicinal plant Bolbostemma paniculatum (Maxim.) Franquet: A comprehensive review
Source: Front Pharmacol. 2022 Sep 7;13:974054. doi: 10.3389/fphar.2022.974054 (PMC9490187; doi:10.3389/fphar.2022.974054)
Supplement: Supplementary file 1 [file Table1.docx]

**Table S1**

**Chemical constituents from *B. paniculatum***

| Classification | No. | Name | Ref. |
| --- | --- | --- | --- |
| Pentacyclic triterpenoids | 1 | Tubeimoside Ⅰ | (Kong et al., 1988a; Tang et al., 2014; Zeng et al., 2018a) |
|  | 2 | Tubeimoside Ⅱ | (Kong et al., 1988b; Tang et al., 2014; Cheng et al., 2006) |
|  | 3 | Tubeimoside Ⅴ | (Tang et al., 2005; Cheng et al., 2006) |
|  | 4 | 6′-*O*-palmitoyltubeimoside I | Liu et al. (2004a) |
|  | 5 | dexylosyltubeimoside Ⅲ | Tang et al. (2015) |
|  | 6 | lobatoside C | Zeng et al. (2018a) |
|  | 7 | Tubeimoside Ⅲ | Kong et al. (1988b)  Tang et al. (2005)  Cheng et al. (2006) |
|  | 8 | actinostemmoside E | Zeng et al. (2018a) |
|  | 9 | actinostemmoside F | Zeng et al. (2018a) |
|  | 10 | actinostemmoside H | Zeng et al. (2018a) |
|  | 11 | cucurbitacin B | (Liu et al., 2004b; Zheng, 2005a) |
|  | 12 | cucurbitacin E | (Tang et al., 2015; Zheng et al., 2007; Liu et al., 2004b) |
|  | 13 | isocucurbitacin B | Tang et al. (2015) |
| Tetracyclic triterpenoids | 14 | Tubeimoside Ⅳ | Kong et al. (1988b)  Cheng et al. (2006) |
|  | 15 | 7β,18,20,26-tetrahydroxy-(20*S*)-dammar-24E-en-3-*O*-α-L-(3-acetyl)arabinopyranosyl-(1→2)-β-D-glucopyranoside | Liu et al. (2004a) |
|  | 16 | 7β,18,20,26-tetrahydroxy-(20*S*)-dammar-24E-en-3-*O*-α-L-(4-acetyl)arabinopyranosyl-(1→2)-β-D-glucopyranoside | Liu et al. (2004a) |
|  | 17 | 7β,18,20,26-tetrahydroxy-(20*S*)-dammar-24E-en-3-*O*-α-L-arabinopyranosyl-(1→2)-β-D-(6-acetyl)-glucopyranoside | Liu et al. (2004a) |
|  | 18 | 7β,20,26-trihydroxy-(20*S*)-dammar-24E-en-3-*O*-α-L-arabinopyranosyl-(1→2)-β-D-glucopyranoside | Liu et al. (2004a) |
|  | 19 | 7β,20,26-trihydroxy-(20*S*)-dammar-24E-en-3-*O*-α-L-(3-acetyl)arabinopyranosyl-(1→2)-β-D-glucopyranoside | Liu et al. (2004a) |
|  | 20 | 7β,20,26-trihydroxy-(20*S*)-dammar-24E-en-3-*O*-α-L-(4-acetyl)arabinopyranosyl-(1→2)-β-D-glucopyranoside | Liu et al. (2004a) |
|  | 21 | 7β,20,26-trihydroxy-8-formyl-(20*S*)-dammar-24E-en-3-*O*-α-L-(3-acetyl)arabinopyranosyl-(1→2)-β-D-glucopyranoside | Liu et al. (2004a) |
|  | 22 | 7β,20,26-trihydroxy-8-formyl-(20*S*)-dammar-24E-en-3-*O*-α-L-(4-acetyl)arabinopyranosyl-(1→2)-β-D-glucopyranoside | Liu et al. (2004a) |
|  | 23 | 23,24-dihydrocucurbitacin E | Tang et al. (2015) |
|  | 24 | 23,24-dihydrocucurbitacin B | Tang et al. (2015) |
|  | 25 | 23,24-dihydroisocucurbitacin B | (Tang et al., 2015; Zeng et al., 2018a) |
|  | 26 | isocucurbitacin D 25-*O*-acetate | (Zheng et al., 2007; Zheng, 2005a) |

**Table 2** (*continued*)

| Classification | No. | Name | Ref. |
| --- | --- | --- | --- |
|  | 27 | 3-*O*-α-L-arabinopyranosyl-(1→2)-β-D-glucopyranosyl-bayogenin-28-*O*-β-D-xylopyranosyl-(1→3)-α-L-rhamnopyranosyl-(1→2)-α-L-arabinopyranosylesterglycosides | Tang et al. (2006) |
|  | 28 | lobatoside A | Tang et al. (2015) |
|  | 29 | lobatoside B | Tang et al. (2015) |
|  | 30 | lobatoside E | Zeng et al. (2018a) |
|  | 31 | lobatoside D | Zeng et al. (2018a) |
|  | 32 | lobatoside F | Zeng et al. (2018a) |
| Sterols | 33 | stigmasta-7,22,25-triene-3-ol | (Liu et al., 2004b; Zheng, 2005a) |
|  | 34 | stigmasta-7,22,25-triene-3-*O*-nonadecanoic acid ester | Liu et al. (2004b) |
|  | 35 | stigmasta-7,22,25-triene-3-*O*-β-D-(6′-palmitoyl)glucopyranoside | Liu et al. (2004b) |
|  | 36 | stigmasta-7,22,25-triene-3-*O*-β-D-glucopyranoside | Liu et al. (2004b) |
|  | 37 | Stigmasterol | Zheng (2005a) |
|  | 38 | daucosterol palmitate | (Zheng, 2005a; Zheng et al., 2005b) |
|  | 39 | daucosterol | (Zheng, 2005a; Zheng et al., 2005b) |
|  | 40 | β-sitosterol | (Zheng, 2005a; Zeng et al., 2018a) |
|  | 41 | β-sitosterol palmitate | (Zheng, 2005a; Zheng et al., 2005b) |
|  | 42 | stigmasta-7,16,25-triene-3-ol | (Fu et al., 1987; Zeng et al., 2018a) |
|  | 43 | stigmasta-7,16,25-triene-3-*O-*β-D-glucopyranoside | Liu et al. (2004b) |
|  | 44 | (3β,22E)-stigmasta-7,22,25-trien-3-yl-β-D-glucopyranoside | Zeng et al. (2018a) |
|  | 45 | uzarigenin-3-β-sophoroside | Zeng et al. (2018a) |
|  | 46 | sileneoside H | Zeng et al. (2018a) |
|  | 47 | integristerone A-25-acetate | Zeng et al. (2018a) |
|  | 48 | 24(28)-dehydromakisterone A | Zeng et al. (2018a) |
|  | 49 | 3-oxo-androsta-1,4-dien-17a′-spiro-2′-3′-oxo-oxetane | Zeng et al. (2018a) |
| Alkaloids | 50 | 4-(2-formyl-5-methoxymethylpyrrol-1-yl)butyric acid methyl ester | (Zeng et al., 2018a; Liu et al., 2003) |
|  | 51 | (E)-*N*-hydroxy phenyl ethyl-3-(4-hydroxy-3-methoxy phenyl) acrylamide | Zeng et al. (2018a) |
|  | 52 | 2-(2-formyl-5-methoxymethylpyrrol-1-yl)-3-phenylpropionic acid methyl ester | Liu et al. (2003) |
|  | 53 | α-methyl pyrrole ketone | Liu et al. (2003) |
| Carbohydrate | 54 | glucose | Zheng (2005a) |
|  | 55 | D-fructose | Zheng (2005a) |
|  | 56 | maltose | Zheng (2005a) |
|  | 57 | sucrose | (Zheng 2005a; Zeng et al. 2018a) |
|  | 58 | stachyose | Zeng et al. (2018a) |
| Glycosides | 59 | Isomaltol mannoside | Zheng (2005a) |
|  | 60 | α-hydroxyacetone glucoside | (Zeng et al., 2018a; Ma et al., 2005) |
|  | 61 | β-D-glucose 2 → 1 β-D-glucoside | Ma et al. (2005) |
|  | 62 | Methyl α-D-fructofuranoside | Zheng (2005a) |
|  | 63 | Methyl β-D-fructofuranoside | Zheng (2005a) |
|  | 64 | n-Butyl-β-D-fructopyranoside | (Zheng 2005a; Zheng et al., 2005b; Zeng et al., 2018a) |

**Table 2**(*continued*)

| Classification | No. | Name | Ref. |
| --- | --- | --- | --- |
| Anthraquinones | 65 | emodin | (Liu et al., 2004b; Zheng, 2005a) |
|  | 66 | Emodinmonomethylether | (Zheng 2005a; Zeng et al., 2018a) |
| Flavonols | 67 | quercitrin | Zeng et al. (2018a) |
|  | 68 | 3-*O*-[β-D-pyranrham-nose-(1-6)-β-D-galactopyranose]-5,7,4′-trihydroxyl flavone | (Zeng et al., 2018a; Xiang et al., 2017) |
|  | 69 | 6-C-glucose-5,7,3′,4′-hydroxy flavone | Li et al. (2016a） |
|  | 70 | quercetin-3-*O*-α-L-arabinopyranoside | Zeng et al. (2018a) |
| Aromatics | 71 | maltol | (Liu et al., 2004b; Fu et al., 1987; Zeng et al., 2018a) |
|  | 72 | 4-Hydroxybenzoic acid | Zheng (2005a) |
|  | 73 | dibutyl phthalate | Zeng et al. (2018a) |
| Aldehyde | 74 | 5-hydroxymethylfurfural | (Zheng, 2005; Zheng et al., 2005b) |
| Fatty acid | 75 | Palmitic acid | (Zheng 2005; Ma et al., 2006) |
|  | 76 | hexadecanoic acid | Zeng et al. (2018a) |
|  | 77 | 9-octadecenamide | Zeng et al. (2018a) |
|  | 78 | D-alanine | Zheng (2005) |
| Nucleoside | 79 | Uridine | Zheng (2005) |
|  | 80 | Thymidine | Zheng (2005) |
|  | 81 | Adenosine | Ma et al. (2006) |
|  | 82 | cytosine | Ma et al. (2006) |
| Alcohols | 83 | D-Sorbitol | Zheng (2005) |
|  | 84 | D-Mannitol | Zheng (2005) |
| Phenols | 85 | chlorogenic acid | (Zeng et al., 2018a; Xiang et al., 2017) |
|  | 86 | pyrogallol | Zeng et al. (2018a) |
|  | 87 | Catechin | Xiang et al. (2017) |
|  | 88 | epicatechin | Xiang et al. (2017) |
| Phenylpropionic acids | 89 | scopoletin | (Zeng et al., 2018a; Xiang et al., 2017) |
|  | 90 | 5-*O*-feruloylquinic acid | Zeng et al. (2018a) |
| Alkane | 91 | n-Hentriacontane | Ma et al. (2005) |
|  | 92 | Nonacosane | Ma et al. (2006) |
|  | 93 | triacontane | Ma et al. (2006) |
| Heterocycle | 94 | allantoin | Ma et al. (2006) |
| Amides | 95 | (*E*)-N-(4-hydroxyphenethyl)-3-(4-hydroxy-3-methoxyphenyl)-acrylamide | Xiang et al. (2017) |
| Lignin | 96 | (*Z*)-3-*O*-caffeoyl-4-*O*-methylquinic acid methyl ester | Xiang et al. (2017) |
