# Supplementary material for: Chinese endemic medicinal plant Bolbostemma paniculatum (Maxim.) Franquet: A comprehensive review
Source: Front Pharmacol. 2022 Sep 7;13:974054. doi: 10.3389/fphar.2022.974054 (PMC9490187; doi:10.3389/fphar.2022.974054)
Supplement: Supplementary file 2 [file Table2.docx]

**Table S2**

**Pharmacological activities of *B. paniculatum***

| Active components | Experimental object/Method | Dose/Duration | Control | | Results | Ref. |
| --- | --- | --- | --- | --- | --- | --- |
|  |  |  | Positive | Negative |  |  |
| Tubeimoside Ⅰ (TBMS Ⅰ) | PC3 cells / *in vitro* | 1, 2.5, 5, 10, 20, 50 μg/mL / 24 h | NA | Blank  medium | TBMS Ⅰ significantly inhibited the proliferation of PC3 cells and had some anti prostate cancer effects. | Wang et al. (2016) |
| TBMS Ⅰ | HepG2, L-02, HCCLM3, QSG-7701 cells / *in vitro* | 5, 10, 15, 20, 30, 40, 90 µM / 24 h | NA | Blank  medium | TBMS Ⅰ potently inhibited growth in HepG2 cells by mediating a cascade of apoptosis signaling pathways. | Wang et al. (2011) |
| TBMS Ⅰ | A2780/DDP cells / *in vitro* | IC_50_ values:  16.10±0.33 μM/L / 12, 24, 48 h | NA | Saline | TBMS I can significantly increase the inhibitory effect of Cisplatin on the proliferation of human ovarian cancer A2780/DDP cells, and cyclin A, cyclin D1 may be involved in its regulation. | Liu et al., (2011) |
| TBMS Ⅰ | NCI-H1299 cells / *in vitro* | 0, 2.5, 5, 10, 25, 50µM / 48 h | 5-Fu | NA | TBMS Ⅰ inhibited the proliferation and metastasis, and promoted the apoptosis of NCI-H1299 cells, which may be mediated by over-expressing miR-126-5p, which inactivates the VEGF‑A/VEGFR2/ERK signaling pathway. | Shi et al., (2018b) |
| TBMS Ⅰ | U87, LN229 cells / *in vitro* | 1, 5, 10, 20 μM/L / 24 h | NA | DMSO | TBMS I could inhibit the proliferation and migration of glioblastoma cells by increasing the ubiquitination level of MET. | Cao et al. ( 2019) |
| Lipid soluble extract of fresh *B. paniculatum* | BT-549,MCF-7,MCF-7/ADR-RES,MDA-MB-231/ATCC cells / *in vitro* | 30 μg/mL / 48 h | Paclitaxel | NA | The lipid soluble fraction of fresh *B. paniculatum* was mainly sterols, which were cytotoxic to four breast cancer cell lines. | Liu et al., (2013b) |
| Petroleum ether-, EtOH-, EtOAc-, n-BuOH-, H2O-soluble extracts | BGC-823, HeLa, HT-29 and MCF-7 cells / *in vitro* | IC_50_values:  0.93~9.73μM  IC_50_values:  1.30~15.64 μM | 5-Fu | NA | The cytotoxic activity of the bulbs of *B. paniculatum* mainly ascribable to cucurbitacine triterpenoid sapogenins and the cyclic bisdesmosides. | Tang et al., (2015) |
| TBMS Ⅰ | NCI-H460 cells / *in vitro* | 20 µM / 9 h | NA | PBS | TBMS Ⅰ-induced NCI-H460 cell cytotoxicity involved nucleolar stress-induced p53/murine double minute clone 2 (MDM2), mTOR, and NF-κB signaling pathways. | Lin et al. (2016) |
| TBMS Ⅰ | HeLa cells / *in vitro* | IC_50_values:  25 μM / 24 h | NA | NA | TBMS Ⅰ exerts cytotoxicity in HeLa cells through both mitochondrial dysfunction and ER stress cell death pathways. | Xu et al. (2009) |

**Table 3** (*continued*)

| Active components | Experimental object/Method | Dose/Duration | Control | | Results | Ref. |
| --- | --- | --- | --- | --- | --- | --- |
|  |  |  | Positive | Negative |  |  |
| TBMS Ⅰ | HeLa cells / *in vitro* | 12.5, 25 µM / 24-72 h | NA | Blank  medium | TBMS Ⅰ induced cell cycle arrest at G2/M phase accompanied by a decrease in G0/G1 phase in HeLa cells; TBMS1 inhibited the levels of cyclinB1, Cdc2 and Cdc25C, but enhanced Chk2 phosphorylation. | Xu et al. (2011) |
| Tubeimoside microcapsule embolization | New Zealand purebred white rabbits, VX_2_ cells / *in vivo* | 1.0 mL / 7 d | NA | Saline, PBS | TBMS microcapsule embolization provides inhibition on tumor growth and VEGF expression and thus be considered as the proper peripheral embolization agent. | Guo et al. (2006) |
| Medicated serum of *B. paniculatum* water decoction | A549, H1299 cells / *in vitro* | 2.5%, 5.0%, 10.0%, 20.0%, 40.0% / 24 h | NA | Blank serum | The proliferation and induction of apoptosis of human lung cancer A549 and H1299 cells were significantly inhibited by the medicated serum of *B. paniculatum*, and the mechanism may be related to the induction of apoptosis by the mitochondrial pathway. | Mi et al. (2021) |
| Peiminine | Hela, HepG2, SW480 and MCF-7 cells / *in vitro* | 0, 2, 4, 6, 8, 10, 12, 14μg/mL / 24, 48, 72 h | NA | Blank  medium | Peiminine can induce apoptosis inhuman hepatocellular carcinoma HepG2 cells through both extrinsic and intrinsic apoptotic pathways. | Chao et al. (2019) |
| TBMS Ⅰ | SKOV-3 cells / *in vitro* | 2, 4, 8, 16µM / 24 h | NA | Blank  medium | TBMS Ⅰ-induced SKOV-3 cell apoptosis correlated with ER stress, increased Bax expression via phosphorylation of p38/MAPK elevation and decreased Bcl-2 levels via phosphorylation of ERK1/2 reduction. | Chen et al. (2012) |
| TBMS Ⅰ | EC109 cells / *in vitro* | 5, 10, 15, 20, 25, 30, 35, 40, 45, 50, 55, 60 μM/L/ 24 h | NA | NA | TBMS Ⅰ-induced apoptosis is through mitochondrial intrinsic apoptosis and P21-cdc2/cyclin B1 signaling pathway. | Xu et al. (2013) |
| TBMS Ⅰ | DU145, PC3 cells / *in vitro* | IC_50_values:  10, 20 μM/L / 24 h | NA | DMSO | TBMS Ⅰ induces oxidative stress-mediated apoptosis in DU145 human prostate cancer cells in vitro via the mitochondrial pathway. | Yang et al. (2016) |
| TBMS Ⅰ | JEG-3 cells / *in vitro* | 1~24μM / 24 h, 48 h | NA | Blank  medium | TBMS Ⅰ is an efficient apoptosis-inducing agent for choriocarcinoma cells, which exerts its effects, at least partially, by the induction of mitochondrial dysfunction and regulation of the p38/MAPK, ERK1/2 and PI3K/Akt signaling pathways. | Huang et al. (2011) |
| TBMS Ⅰ | CNE-2Z cells / *in vitro* | IC_50_ values:  16.7, 20.7,32.5,µM/L/24,48,72h | NA | Blank  medium | TBMS Ⅰ can induce the apoptosis of CNE-2Z cells, and the induction of apoptosis by TBMSⅠ is closely associated with down-regulation and phosphorylation of Bcl-2 and Bax activation. | Weng et al. (2003) |

**Table 3** (*continued*)

| Active components | Experimental object/Method | Dose/Duration | Control | | Results | Ref. |
| --- | --- | --- | --- | --- | --- | --- |
|  |  |  | Positive | Negative |  |  |
| TBMS Ⅰ | A549cells / *in vitro* | 2,4,8,12,16µM/L/24,48,72h | NA | Blank  medium | TBMS Ⅰ treatment induced apoptosis by decreasing the expression of Bcl-2 and increasing the expression of Bax in the A549 cells. | Zhang et al. (2011a) |
| TBMS Ⅰ | A549, PC9 cells / *in vitro* | 0, 4, 8, 12, 16, 20, 24, 28, 32 μM/L / 72 h | NA | NA | TBMS Ⅰ can inhibit the in vitro growth, arrest the cell cycle and induce the apoptosis of lung cancer cells, its mechanisms is related to the regulation of associated protein expression, activation of MAPK-JNK signaling pathway. | Hao et al. (2015) |
| TBMS Ⅰ | BGC823 cells / *in vitro* | 0, 5, 10, 15, 20µM/L / 24, 48 h | NA | Blank  medium | TBMS Ⅰ inhibited proliferation and promoted apoptosis in BGC823 gastric cancer cells,this apoptotic response is associated with the regulation of the expression of the Bcl-2 gene family. | Zhang et al. (2013) |
| TBMS Ⅰ | U251 cells / *in vitro* | 0‑50µg/mL, 30µg/mL / 24, 48, 72 h | NA | DMSO | TBMS Ⅰ prevented the progression of gliomas via the PI3k/Akt-dependent pathway. | Cao et al. (2020) |
| TBMS Ⅰ | U251cells / *in vitro* | 10, 15, 20, 25, 30, 35, 40, 45, 50 μg/mL / 24h, 48h, 72h | NA | DMSO | TBMS Ⅰ induced apoptosis by increasing the concentration of reactive oxygen species through the release of Cytochrome C and activation of Caspase-3. | Jia et al. (2015) |
| TBMS Ⅰ, TBMSⅢ | SW480 cells / *in vitro* | 3.14, 4.05 mg/L / 3 d | NA | Blank  medium | TBMS I and TBMS III induced apoptosis in human rectal cancer SW480 cells. | Yu et al. (2006) |
| *B. Paniculatum* preparation | Tca8113 cells/ *in vitro* | 40 μg/mL / 12 h | NA | Blank  medium | Treatment with the combination of *B. paniculatum* preparation and hyperthermia increased the rate of inhibition of cell proliferation, inhibited the progression of G1 to S phase transition, increased apoptosis rate and sub-cellular structural changes in Tca8113 cells. | Han et al. (2009) |
| TBMS Ⅰ | HL-60 cells / *in vitro* | 10,20,30,40μM/L / 24, 48, 72 h | NA | Blank  medium | TBMS I arrested the cell cycle of human myeloid leukemia cell HL-60 and induced its apoptosis, and the mechanism may be related to decreased cyclin B1 expression. | Hu et al. (2003b) |
| TBMS Ⅰ | HeLa cells / *in vitro* | 20, 40, 80 μM/L, 10 μM/L / 24 h | NA | Blank  medium | TBMS Ⅰ and Cisplatin alone and in combination can block the cell cycle in G2/M phase of cervical cancer HeLa cell line. | Zhang et al. (2010) |
| TBMS Ⅱ | HepG2 cells, H22 mice/ *in vitro and vivo* | IC_50_ values:  4.05 μg/mL / 24 h, 5, 10 mg/kg / 14 d | NA | NA, Sterile water | TBMS Ⅱ can inhibit cell proliferation probably via promoting tumor cells to stagnate in G2/M phase. | Chao et al. (2012) |

**Table 3** (*continued*)

| Active components | Experimental object/Method | Dose/Duration | Control | | Results | Ref. |
| --- | --- | --- | --- | --- | --- | --- |
|  |  |  | Positive | Negative |  |  |
| Dichloromethane extract of fresh *B. paniculatum* | MDA-MB-231-GFP cells / *in vivo* | 50, 75 mg/kg / 58 d | NA | NaCl | Dichloromethane extract of fresh *B. paniculatum* showed inhibitory effects on tumor growth and the development of lung metastasis in a nude mouse model of human triple negative breast cancer MDA-MB-231-GFP. | Bao et al. (2017) |
| Dichloromethane extract of fresh *B. paniculatum* | MDA-MB-231-GFP cells */ in vitro* | 1.875, 3.75, 7.5, 15,30,60,120, 480μg/mL / 24, 48 h | NA | DMSO, NaCl | Dichloromethane extract of fresh *B. paniculatum* has anti-metastatic effects on MDA-MB-231-GFP tumor. | An et al. (2013) |
| Total saponins of *B. paniculatum* | MDA-MB-231 cells / *in vitro* | IC_50_ values:  10μg/mL / 48 h | NA | NA | Total saponins of *B. paniculatum* decreased proliferation and migration of MDA-MB-231 cells possibly through inhibiting the PI3K/Akt/mTOR signaling pathway. | Dou et al. (2019) |
| TBMS Ⅰ | MDA-MB-231 cells / *in vivo and vitro* | 5μM / 24 h | NA | DMSO | TBMS Ⅰ suppressed the CXCR4-mediated metastasis of breast cancer by inhibiting NF-κB binding activity. | Peng et al. (2016) |
| TBMS Ⅰ | SW480, HCT-8 cells / *in vitro* | 0, 10, 20, 50µg/ml / 24 h | NA | Blank  medium | TBMSI inhibited CRC cell proliferation and invasion via suppressing the Wnt/β-catenin signaling pathway. | Bian et al. (2015) |
| TBMS Ⅰ | NCI-H1299  cells / *in vitro* | 0, 2.5, 5, 10, 25, 50µM / 48 h | 5-Fu | Blank  medium | TBMSI increases miR-126-5p expression, whereas over-expressing miR-126-5p inactivates VEGF-A/VEGFR-2/ERK signaling pathway, which ultimately actuates the pro-apoptotic and anti-metastatic effects in NCI‑H1299 cells. | Shi et al. (2018a) |
| TBMS Ⅰ | PGCL3 cells / *in vitro* | 5.0, 10.0, 15.0 , 20.0 μM/L / 24, 48, 72 h | NA | RPMI-1640 | TBMS I inhibits the adhesion, invasion, and migration of human high metastatic giant cell lung cancer PGCL3 cells, the effect may be related to the decreased secretion of MMP-2 and its activity in PGCL3 cells, the reduced adhesion of PGCL3 cells to laminin and fibronectin. | Yu et al. (2008a) |
| TBMS Ⅰ | HepG2 cells / *in vitro* | 10, 20, 30μM/L / 8 h | NA | Blank  medium | TBMS I is able to inhibit the migration and invasion of HepG2 cells *in vitro*, and the possible mechanism may relate to inhibitory effects on the activities and expressions of MMP-2 and MMP-9. | Zhong et al. (2016) |

**Table 3** (*continued*)

| Active components | Experimental object/Method | Dose/Duration | Control | | Results | Ref. |
| --- | --- | --- | --- | --- | --- | --- |
|  |  |  | Positive | Negative |  |  |
| TBMS Ⅰ | SCL-1 cells / *in vitro* | 5, 10, 20 μg/mL / 24 h | NA | Blank  medium | TBMS I may inhibit the proliferation, migration and invasion of skin squamous cell carcinoma cells and promote cells apoptosis by regulating circ_0000376 /miR-203 axis． | Wang et al. (2021) |
| TBMS Ⅰ | B16, Lewis cells and BALB/c nude mice / *in vivo* | 2, 3 mg/kg/d, 2 mL / 15 d, 21 d | Cyclophosphamide | Saline | TBMS I significantly inhibited both experimental metastasis of murine B16 melanoma and spontaneous metastasis of Lewis lung carcinoma. | Wang et al. (2006) |
| TBMS Ⅱ | Y-79, WERI-Rb-1 cells / *in vitro* | 0, 1, 2.5, 5 μM / 48 h | NA | NA | TBMS II exerted its inhibitory effect against TGF-β1-induced metastatic progression of Rb cells via suppressing redoxosome-dependent EGFR activation including EGFR phosphorylation and oxidation. | Chen et al. (2021) |
| TBMS Ⅰ | Chicken embryos / *in vitro* | 5, 10, 20, 30, 40, 50 μmol/L / 3d | NA | PBS | TBMS Ⅰ has an obvious inhibitory effect on angiogenesis, and the dose-effect relationship is quite distinct. | Hu et al. (2003a) |
| TBMS Ⅰ | HUVECs, BALB/c nu/nu mice / *in vivo and vitro* | 10, 15, 20, 25, 30 μmol/L / 24, 48, 72 h, 14 d | NA | PBS | TBMS Ⅰ can significantly inhibited angiogenic activity, and its inhibitory effect was associated with inducing apoptosis of vascular endothelial cells, inhibiting their motility, and down-regulating the expression of VEGF, bFGF, and PDGF. | Yu et al. (2008b) |
| TBMS Ⅰ | CD1 nu/nu mice, eEND2, NSCLC, NCI-H460, A549 cells / *in vivo and vitro* | 5 mg/kg, 2.5~50 μM / 17 d, 24 h | NA | 0.9% NaCl | TBMS Ⅰ inhibits angiogenesis, which is based on its stimulatory action on proteasomal VEGFR2 and Tie2 degradation. | Gu et al. (2016) |
| TBMS Ⅰ | eEND2,HDEMC,NCI-H460,A549 cells / *in vivo and vitro* | 5 mg/kg, 2.5, 5, 10, 25, 50 μM/L / 17 d, 24 h | NA | Saline | TBMS I can inhibit tumor angiogenesis, and its mechanism of action may be related to down-regulation of VEGF/VEGFR2 and Ang2/Tie2 signaling path． | Li et al. (2016b) |
| TBMS Ⅰ | HUVECs, C57BL/6 mice / *in vivo and vitro* | 5μM, 10μM / 30 min | NA | NA | We speculate that the anti-angiogenic effect of TBMS I may be related to the Piezo1 channel. | Liu et al. (2020) |

**Table 3** (*continued*)

| Active components | Experimental object/Method | Dose/Duration | Control | | Results | Ref. |
| --- | --- | --- | --- | --- | --- | --- |
|  |  |  | Positive | Negative |  |  |
| TBMS Ⅰ | MDA-MB- 231 cells / *in vitro* | 5, 10, 15, 20, 25, 30μg/mL / 48 h | NA | Blank  medium | TBMS Ⅰ inhibit the proliferation, promote apoptosis and induce autophagy in breast cancer cell line MDA-MB-231, the autophagy induction effect of TBMSⅠ may be realized by regulating the PI3k-Akt-mTOR signaling pathway. | Liu et al. (2019a) |
| TBMS Ⅰ | HepG2 cells / *in vitro* | 0, 0.4, 1, 2, 4, 8, 12, 16 μM / 24 h | NA | NA | TBMS Ⅰ triggered autophagy in HepG2 cells by inducing the accumulation of impaired autophagosomes, and its mechanisms of action may be associated with the activation of the AMPK signaling pathway. | Ruan et al. (2020) |
| TBMS Ⅰ | SW480, SW620 cells / *in vitro* | 0, 5, 10, 15, 20, 25, 30 μM / 24 h | NA | NA | TBMS Ⅰ induces CRC cell death by inducing ROS-induced impaired autophagolysosomes accumulation. | Yan et al. (2019) |
| TBMS Ⅰ | BALB/c nude mice, HeLa, SiHa cells / *in vivo and vitro* | 3 mg/kg/d, 15 µM / 16 d, 24 h | NA | Saline | TBM Ⅰ triggers cell death and promotes CDDP sensitivity in cervical cancer by inducing the accumulation of impaired autophagolysosomes. | Feng et al. (2018) |
| TBMS Ⅰ | NCI-H1299, NCI-H1975 cells / *in vitro* | 0, 5, 10, 15, 20, 25, 30, 35, 40, 45 µM / 24 h | NA | NA | TBMS Ⅰ exerts a dual anticancer effect that involves the disruption of mitochondrial and lysosomal pathways and their interaction and, thereby, has a specifific and enhanced killing effect on lung cancer cells. | Wang et al. (2020) |
| TBMS Ⅰ | A375, MV3 cells / *in vitro* | 4,8,12µM, 6,12,18µM/48h | NA | DMSO | TBMS Ⅰ might activate PTP1B, which further hyper-activates MEK1/2-ERK1/2 cascade, thereby inhibiting cell proliferation in melanoma. | Du et al. (2020) |
| Tubeimosides | Long ear white rabbits / *in vivo* | 500 μL/d / 20 d | NA | NA | Tubeimosides has a certain curative effect in the treatment of experimental HSK, and the combined application of bFGF can enhance its curative effect in the treatment of HSK. | Zhang et al. (2011b) |
| Tubeimosides | Ducks */ in vivo* | 0.01, 0.1, 1 mg/kg/d / 10 d | Lamivudine (3TC) | Saline | TBMS can reduce the level of DHBV-DNA in duck serum. | Zhou et al. (2007) |
| Tubeimosides | HepG_2_2.2.15 cells / *in vitro* | 0.01, 0.1, 1, 5, 10 μg/mL / 24 h, 48 h, 72 h | 3TC | Blank  medium | In vitro cell culture showed that Tubeimosides inhibited HBV. | Zhou and Wu (2005) |
| TBMS Ⅰ | Vero cells, HSV-1 F lines / *in vitro* | 0.39, 0.78, 1.56, 3.13, 6.25, 12.50, 25.00, 50.00, 100.00 μg/mL / 24, 48, 72 h | Aciclovir | Blank  medium | TBMS I showed significant activity in inhibiting the proliferation and genomic replication of herpes simplex virus type 1 and 2, and a nontoxic high concentration of aglycone also showed inhibitory activity against the proliferation of HSV-1/106 drug-resistant strain. | Wang et al. (2019) |

**Table 3** (*continued*)

| Active components | Experimental object/Method | Dose/Duration | Control | | Results | Ref. |
| --- | --- | --- | --- | --- | --- | --- |
|  |  |  | Positive | Negative |  |  |
| TBMS Ⅰ | HEK293T, Vero, Vero E6, A549, Caco-2 cells / *in vitro* | 10 µM / 2 h | NA | NA | We identified TBMS Ⅰ and nigericin sodium as novel compounds which exhibited potent antiviral activities against authentic SARS-CoV-2 infection in vitro. | Ju et al. (2021) |
| Tubeimosides | Hartley guinea pigs / *in vivo* | 1, 5, 10, 20mg/ml / 28 d | NA | Ionic, distilled water | Tubeimosides have obvious activity against the delayed contact hypersensitivity induced by PPDA in guinea pigs. | Yu et al. (2016) |
| TBMS Ⅰ | ICR rats/ *in vivo and vitro* | 10, 50, 100 μg/ a ear, 5, 10, 15 μg/mL / 5 h、48 h | NA | Acetone, Blank  medium | TBMS I was performed at the dose of 100μg/ear and 50μg/ear significantly inhibited TPA and AA induced edema in the murine ear, it also exhibited anti carcinogenic effects. | Ma et al. (1991) |
| Nbutyl alcohol fraction of *Smilax glabra* and *B. paniculatum* | SD rats / *in vivo* | 346.5 mg/kg / 7 d | Dexamethasone | Saline | The n-butyl alcohol fraction of *Smilax glabra* and *B. paniculatum*  possesses significant anti-RA and anti-inflammatory effects, and it has a potential to be developed as a new therapeutic agent against RA. | Bao et al. (2018) |
| TBMS Ⅰ | Wistar rats, FLS cells / *in vivo and vitro* | 1, 5, 10 mg/kg/d, 0, 1, 2.5, 5, 10 µM / 14 d、24 h | NA | Saline | TBMS I suppressed TNF α-induced activations of NF-κB and MAPKs (p38 and JNK) leading to the down-regulation of pro-inflammatory cytokines, which was beneficial to the anti-proliferative and anti-migratory activities of FLS cells. | Liu et al. (2018) |
| TBMS Ⅰ | Male BALB/c mice / *in vivo* | 45, 90, 180 mg/kg / 21 d | NA | PBS | TBMS Ⅰ treatment may ameliorate PM2.5-induced damage by suppressing inflammation. | Zhang et al. (2018) |
| TBMS Ⅰ | BALB/c mice, RAW 264.7 cells */ in vivo and vitro* | 1, 2, 4 mg/kg, 2, 4, 6 µM / 7 h、24 h | NA | PBS | TBMS Ⅰ inhibits inflammation both in vitro and in vivo, and may be a potential therapeutic candidate for the prevention of inflammatory diseases. | Zhang et al. (2018) |
| TBMS Ⅰ | Adult male Wistar rats, BV-2 cells / *in vivo and vitro* | 1, 2, 4 mg/kg/d, 0, 1, 2, 4, 8 µM / 24 d, 24 h | NA | NA | TBMS I played a role in protecting dopaminergic neurons by inhibiting neuroinflammation mediated by microglia. | He et al. (2018) |
| TBMS Ⅰ | Vascular smooth muscle cells, wild-type or iNOS−/− C57BL/6 mice / *in vivo and vitro* | 1 mg/kg/d / 7 d | NA | Saline | TBMS I prevented iNOS expression by inhibiting TLR4-MyD88-NF-κB pathway, TBM enhanced vascular responsiveness to NE and KCl, partially restored the MAP and at last improved survival of septic mice. | Luo et al. (2020) |

**Table 3** (*continued*)

| Active components | Experimental object/Method | Dose/Duration | Control | | Results | Ref. |
| --- | --- | --- | --- | --- | --- | --- |
|  |  |  | Positive | Negative |  |  |
| TBMS Ⅰ | H9c2 cells, wild-type male C57BL/6 mice / *in vivo and vitro* | 0~4μM,4 mg/kg / 12h, 18 h | NA | NA | TBMS Ⅰ protects against sepsis-induced cardiac dysfunction by reducing inflammation, oxidative stress and apoptosis via SIRT3. | Cheng et al. (2021) |
| TBMS Ⅰ | HUVECs cells, wild-type male C57BL/6 mice /*in vivo and vitro* | 0~2 μM / 12, 24 h | NA | NA | TBMS Ⅰ protects against sepsis-induced endothelial dysfunction by reducing oxidative stress and apoptosis via SIRT3. | Yang et al. (2021) |
| TBMS Ⅰ | Human K562 cells, ICR mice / *in vitro* | 25, 50, 100 μg / 14d | Quil A | PBS | TBMS I has immune adjuvant activity against ovalbumin (OVA), which can promote antigen-specific humoral and cellular immune responses in immunized mice, and can simultaneously induce Th1 / Th2 immune responses. | Han (2019) |
| TBMS Ⅰ | H460, H157, A375, A2058 cells, C57BL/6, BALB/c nude mice/ *in vivo and vitro* | 5 µM/L, 0, 1, 2, 4, 8, 16 mg/kg/d / 24 h, 14 d | NA | NA | TBMS Ⅰ, by virtue of its ability to bind to and subsequently inactivate mTOR, induces TFEB-dependent lysosomal degradation of PD-L1 in cancer cells and thus ameliorates the immunosuppressive tumor micro-environment and improves anti-tumor T-cell immunity. | Liu et al. (2021) |
| TBMS Ⅰ | Mice / *in vivo* | 0.2 mL/d / 7 d | NA | Saline | TBMS I significantly inhibits hemolysin production and delayed hypersensitivity in mice. | Huang et al. (1992) |
| TBMS Ⅰ | BALB/c mice, splenocytes / *in vivo and vitro* | 0-12 µM, 2, 4, 6 µM / 48 h | NA | Blank  medium | Administration of TBMS I significantly inhibited T cell-mediated DTH response *in vivo*, the mechanism correlated with the suppressing activation of NF-kB, NFAT2 and AP-1 signal transduction pathways. | Huang et al. (2015) |
| TBMS Ⅰ | C57BL/6 mice / *in vivo* | 0.5, 1,2μM/L / 5 d | RANKL | Blank  medium | TBMS I exerts a protective effect on osteoporosis in OVX mice by inhibiting the differentiation of osteoclasts, and the mechanism may be related to the inhibition of NF-κB signaling pathways. | Yang et al. (2019) |
| TBMS Ⅰ | BMM cells, C57BL/6 mice / *in vitro* | 50 ng/ml / 5 d | RANKL | Blank  medium | TBMS I attenuates osteoclastogenesis through down-regulating NF-κB signaling pathway. | Yang et al. (2020) |

**Table 3** (*continued*)

| Active components | Experimental object/Method | Dose/Duration | Control | | Results | Ref. |
| --- | --- | --- | --- | --- | --- | --- |
|  |  |  | Positive | Negative |  |  |
| 75% ethanol extract of *B. paniculatum* | Escherichia coli, Salmonella, Streptococcus agalactiae, Staphylococcus aureus / *in vitro* | 0.5 g/ml / 24h | Amikacin sulfate | NA | After mixing the equal ratio of *B. paniculatum* and Angelica dahurica extract, which complemented the deficiency of the two drugs in the inhibition of bacteria, and the inhibitory effects on the above four bacteria have reached the level of medium sensitivity or above. | Chen et al. (2009) |
| Total saponins of *B. paniculatum* and TBMS I, Ⅳ | Semen from healthy men / *in vitro* | 0.05, 0.1, 1% / 1, 3, 5, 10, 20, 30 min | NA | NA | Total saponins of *B. paniculatum* and TBMS I, TBMS Ⅳ all showed potent spermicidal effects, the mechanism being mainly disruption of the biofilm system of spermatozoa. | Su and Guo (1986) |
| TBMS Ⅰ | C57BL/6 mice, H9c2 cells / *in vivo and vitro* | 4mg/kg / 24h, 3h | NA | NA | TBMS Ⅰ protects against MIRI through SIRT3-dependent regulation of oxidative stress and apoptosis. | Lv et al. (2021) |
